# Rewritable Triple-Mode Light-Emitting Display

**DOI:** 10.1007/s40820-025-01686-4

**Published:** 2025-03-13

**Authors:** Seokyeong Lee, Jong Woong Park, Jihye Jang, Jin Woo Oh, Gwanho Kim, Jioh Yoo, Jong Gun Jung, Hyowon Han, Wei Jiang, Chang Eun Lee, Jungwon Yoon, Kaiying Zhao, Cheolmin Park

**Affiliations:** 1 Department of Materials Science and Engineering, Yonsei University, Seoul, 03722 Republic of Korea; 2 Post-Silicon Semiconductor Institute, Korea Institute of Science and Technology, Seoul, 02792 Republic of Korea

**Keywords:** Alternating-current electroluminescence, Room-temperature phosphorescence, Stimuli-responsive materials, Multimode luminescence, Information encryption

## Abstract

Despite great progress in developing mode-selective light emission technologies based on self-emitting materials, few rewritable displays with mode-selective multiple light emissions have been demonstrated. Herein, we present a rewritable triple-mode light-emitting display enabled by stimuli-interactive fluorescence (FL), room-temperature phosphorescence (RTP), and electroluminescence (EL). The display comprises coplanar electrodes separated by a gap, a polymer composite with FL inorganic phosphors (EL/FL layer), and a polymer composite with solvent-responsive RTP additives (RTP layer). Upon 254 nm UV exposure, a dual-mode emission of RTP and FL occurs from the RTP and EL/FL layers, respectively. When a polar liquid, besides water, is applied on the display and an AC field is applied between the coplanar electrodes, EL from the EL/FL layer is triggered, and the display operates in a triple mode. Interestingly, when water is applied to the display, the RTP mode is deactivated, rendering the display to operate in a dual mode of FL and EL. By manipulating the evaporation of the applied polar liquids and water, the mode-selective light emission of FL, RTP, and EL is rewritable in the triple-mode display. Additionally, a high-security full-color information encryption display is demonstrated, wherein the information of digital numbers, letters, and Morse code encoded in one optical mode is only deciphered when properly matched with that encoded in the other two modes. Thus, this article outlines a strategy to fulfill the substantial demand for high-security personalized information based on room-temperature multi-light-emitting displays.

**Supplementary Information:**

The online version contains supplementary material available at 10.1007/s40820-025-01686-4.

## Highlights

- A rewritable triple-mode light-emitting display (RE-TriLED) was fabricated, enabled by stimuli-interactive fluorescence (FL), room-temperature phosphorescence (RTP), and electroluminescence (EL).
- Mode-selective multiple light emission is achieved, in which the three emission modes of FL, RTP, and EL are readily manipulated through the controlled evaporation of polar liquids and water.
- A high-security, full-color information encryption display is demonstrated, wherein optical information encoded in one mode is decipherable only when properly aligned with the other two modes.

**Supplementary Information:**

The online version contains supplementary material available at 10.1007/s40820-025-01686-4.

## Graphical abstract

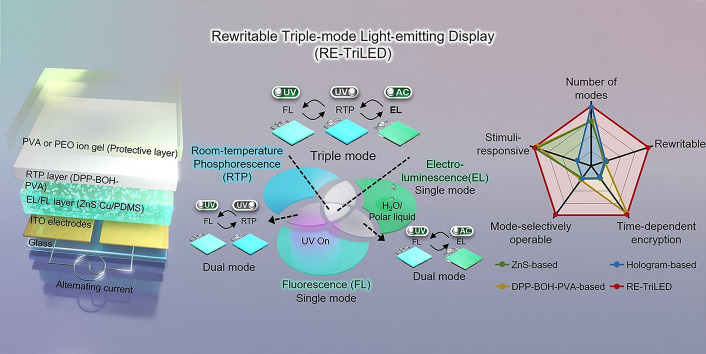

## Introduction

Photoluminescent materials that emit either fluorescence (FL) or room-temperature phosphorescence (RTP) are of great interest in broad scientific and technological fields with numerous applications such as optical sensors, biomarkers, encryptions, and anticounterfeits [1, 2]. Development of materials capable of emitting both FL and RTP broadened their usages allowing more complex and switchable optical applications in diverse fields, ranging from military to banknotes and cutting-edge products [3–5]. Particularly, numerous FL and RTP encryptions have been performed based on the stimuli-interactive FL with a fast response and short decay time [6–11], and RTP with long emission lifetimes and rich excited-optical states upon UV exposure [4, 12–15].

By combining such photoluminescent FL and/or RTP with electroluminescence (EL) which occurs with electrical inputs such as voltage and current, a system can be more useful since various cutting-edge display technologies such as high-resolution pixel array fabrication, large area micropatterning, and electric circuit design are readily employed [16–19]. An EL display with dual-mode light emission capability can be developed by employing an EL material that in addition emits either FL or RTP. For instance, by manipulating both electrical and optical inputs (mostly UV exposure), a display with EL and FL emitters exhibits the mode-selective light emission of EL, FL, and EL/FL [16]. Great care, however, should be taken to develop a triple-mode display where FL, RTP, and EL can interplay with each other because of the difficulty in manipulating the reversible mode switching between FL and RTP [5]. Moreover, to substantially enhance the versatility of the technology with a simple device architecture, a rewritable display should be developed in which EL is interplayed with the rewritable state of either FL or RTP, allowing the facile control of the number of FL, RTP, and EL modes. Motivated by RTP materials that respond to a wide range of stimuli, such as water and heat [20, 21], we envisioned that a rewritable triple-mode EL display could be realized when EL is manipulated with such stimuli-responsive RTP materials.

**Fig. 1:**
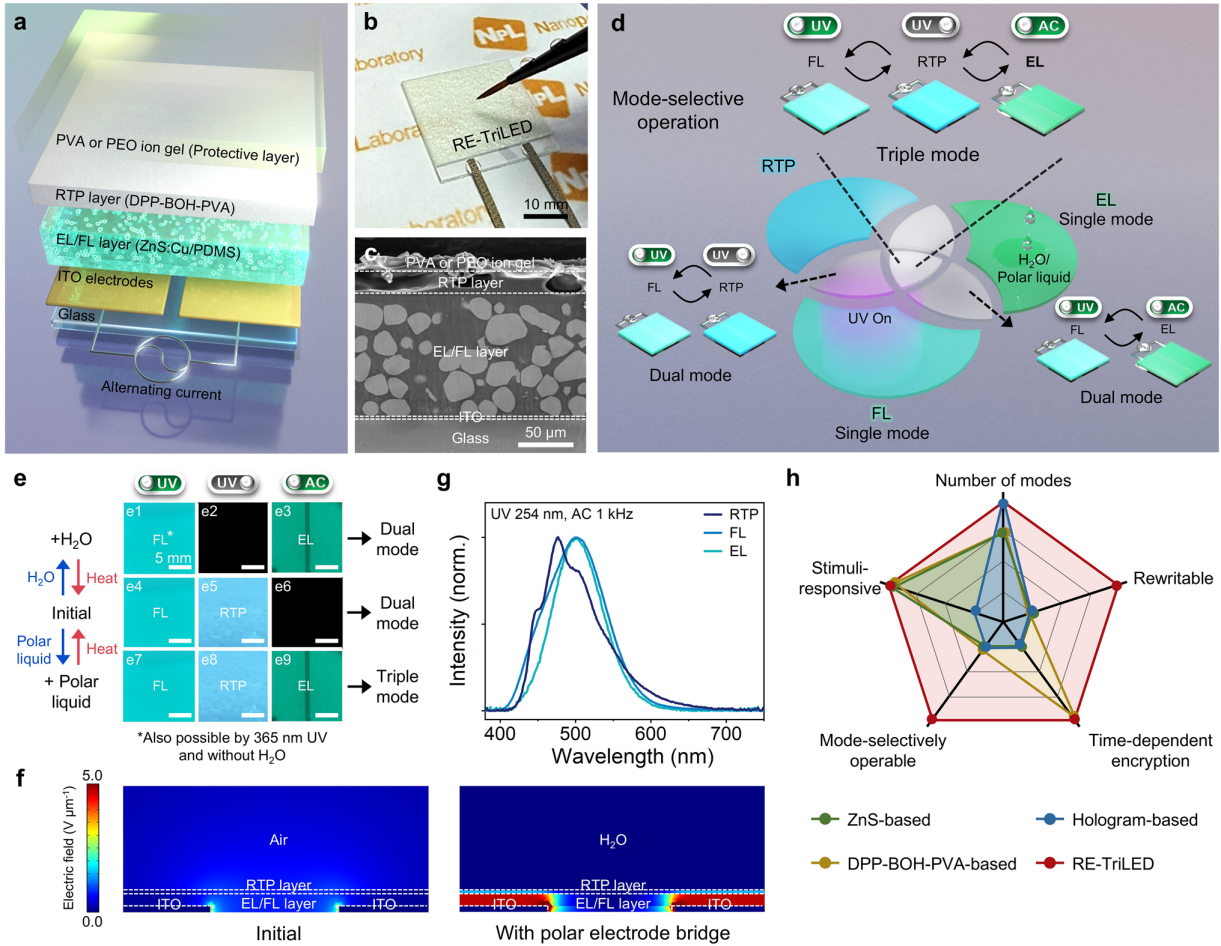
Device architecture and working principles of RE-TriLED. **a** Exploded view layout of RE-TriLED. **b** Photograph of a RE-TriLED together with a polar liquid-soaked brush.** c** Cross-sectional SEM image of a RE-TriLED. **d** Mode-selective operation of the RE-TriLED and its possible emission modes: fluorescence (FL), room-temperature phosphorescence (RTP), and electroluminescence (EL). **e** Photographs of the emission modes of the RE-TriLED upon exposure to UV (254 nm) and AC field at its initial state (**e4**–**e6**), with H_2_O (**e1**–**e3**), and with polar liquid (**e7**–**e9**). **f** Numerical simulations of electric field distribution of RE-TriLED with (right) and without (left) a polar electrode bridge (H_2_O). **g** Normalized spectra of RTP, FL, and EL emissions. **h** Radial plot comparing the different characteristics of the light-emitting optical encryption platforms (see Table S2)

In this work, we present a rewritable mode-selective light-emitting display in which the three emission modes of FL, RTP, and EL are readily manipulated in a reversible manner. Our rewritable triple-mode light-emitting display (RE-TriLED) consists of coplanar electrodes separated by a gap on which a polymer composite with FL phosphors (EL/FL layer) and a composite with phosphorescent chromophores (RTP layer) are sequentially stacked, as schematically shown in Fig. 1a. When an alternating current (AC) field is applied between the coplanar electrodes after a polar electrode bridge is formed on the RTP layer with a polar liquid (besides water) [22], field-induced EL from the EL/FL layer is produced in addition to RTP from the RTP layer. Interestingly, when water is utilized, RTP emission from the RTP layer disappears, leading to the reduction of emission modes. Because FL from the EL/FL layer under UV exposure is maintained in all cases, our device becomes a triple-mode of FL, RTP, and EL light emissions with the use of polar liquids, and the number of modes is readily regulated with the choice of either water or polar liquids. The system is reset by evaporating the applied polar liquid with heat treatment, making the device ready for rewriting new information. High-security full-color encryption is demonstrated with a RE-TriLED in which real Morse code information encoded by RTP is decoded only when correctly integrated with FL and EL. Our methodology represents a substantial advancement in the application of FL, RTP, and EL emissions in a single architecture, further enhancing the capabilities of multi-mode display technology.

## Experimental Section

### Materials

Green fluorescent phosphor microparticles (ZnS:Cu, LP-6844, M-18705-1) were purchased from Lonco Co. LTD, Hong Kong. Polydimethylsiloxane (PDMS) and curing agent (Sylgard 184) were purchased from Dow Corning. 5ʹ-m-terphenylboronic acid (DPP-BOH) was purchased from Tokyo chemical industry Co. LTD. Acetone (≥ 99.5%) and isopropyl alcohol (IPA, ≥ 94.0%) were purchased from Duksan reagents. All other materials, including deionized water (H_2_O), poly(vinyl alcohol) (PVA, M_w_ = 130,000 g mol^−1^), ammonium hydroxide solution, fluorescein, rhodamine B, poly(ethylene oxide) (PEO, *M*_w_ = 5,000,000 g mol^−1^), acetonitrile (anhydrous, 99.8%), 1-ethyl-3-methylimidazolium bis(trifluoromethylsulfonyl)imide (EMI-TFSI, ≥ 98%), ethyl alcohol (EtOH, ≥ 99.5%), ethylene glycol (EG, anhydrous, 99.8%), 1-butanol (99.8%), chloroform (≥ 99.5%), dichloromethane (≥ 99.8%), diethyl ether (anhydrous, ≥ 99.7%), ethyl acetate (anhydrous, 99.8%), and 2-methoxyethanol (anhydrous, 99.8%), were purchased from Sigma-Aldrich and were used as received.

### Preparation of RTP Solution

PVA was stirred in water (12 wt%) at 68 °C for 3 h. DPP-BOH (5 mg) was dispersed in water (4 mL) by adding ammonium hydroxide solution (1 mL) and sonicating the solution in an ultrasonic bath for 20 s. The PVA and DPP-BOH solutions were mixed in a volume ratio of 1:1, 2.5 mL each. After 1 min of vigorous stirring, the mixture was placed in an ultrasonic bath for 1 min and left alone for 5 min to remove the bubbles. For the fabrication of fluorescein-doped RTP (RTP-G) and rhodamine B-doped RTP (RTP-R) films, 0.5 mg of fluorescein or rhodamine B was added to the DPP-BOH solution. Here, 0.5 mL of both PVA and DPP-BOH solutions was additionally used, while the ratio was kept equal (3 mL each was used for the mixture).

### Fabrication of RE-TriLED

Two in-plane indium tin oxide (ITO) electrodes with a 1 mm-sized gap were sputtered onto a 25 mm × 25 mm glass substrate. For interdigitated ITO electrodes with a finger width of 0.5 mm and gap of 0.3 mm, a patterned metal mask was used for the sputtering process. The thickness and sheet resistance of the ITO electrodes were 80 nm and 20 Ω cm^–2^, respectively. The ITO-patterned substrates were sequentially cleaned with acetone and IPA in an ultrasonic bath for 10 min each and then dried in an oven at 65 °C. For the EL/FL layer, a ZnS:Cu/PDMS composite was prepared by mixing ZnS:Cu powder, PDMS liquid, and curing agent with a weight ratio of 20:10:1. The mixture was spin coated on the patterned substrates at 1,000 rpm for 60 s, which were then annealed at 80 °C for 8 h. Agglomerates were not observed when the ZnS:Cu microparticles were combined with PDMS, and the spin-coated ZnS:Cu/PDMS composite emitted uniform EL and FL emissions. Next, 2.5 mL of the as-prepared RTP solution was spray coated on the EL/FL layer at 120 °C using an airbrush (SPARMAX, GP-70), forming the RTP layer. For different types of RTP layers, as-prepared RTP-G or RTP-R solutions were used for the spray coating. When fabricating RE-TriLEDs with a patterned RTP layer, a patterned mask with digit “8” was used for the spray coating process. The PVA layer was fabricated by spin coating the as-prepared PVA solution at 1,000 rpm for 60 s and annealed at 110 °C for 10 min. For the PEO ion gel layer, a 2.5 wt% PEO solution in acetonitrile was prepared by mixing PEO and ionic liquid (EMI-TFSI) with a weight ratio of 1:3 and stirring it for 8 h. The solution was then spin coated on the RTP layer at 1,000 rpm for 60 s and was annealed at 80 °C for 10 min. Rewritable and mode-selective properties of the RE-TriLEDs were achieved by heating the display at 120 °C for 5 min. The RE-TriLEDs were operated at an alternating current (AC) frequency and voltage of 1 kHz and 175 V, respectively.

### Fabrication of RE-TriLED Array

5 × 5 array of ITO electrodes (pixel size, 7 mm × 7 mm) were sputtered onto a 50 mm × 50 mm glass substrate. Because all the electrode arrays were connected, control of the EL emission of the RE-TriLED array was possible with a single AC source. The specific preparation and fabrication conditions of the EL/FL layer of the RE-TriLED arrays were equivalent to those used for the RE-TriLED. The RTP solution was spray coated on selected pixels of the EL/FL layer at 120 °C using an airbrush and mask with 5 × 5 circular patterns (diameter, 7 mm), forming the RTP layer. Each pixel was then covered with either a PDMS layer, PVA layer, or PEO ion gel layer to form circular patches (diameter, 7 mm) by drop casting the as-prepared solutions, which would act as protective layers. The specific preparation conditions of the solutions were equivalent to those used for the RE-TriLED. The emission modes of each pixel were precisely controlled by either fuming the display with water or using a water-soaked brush. Rewritability was achieved by detaching the RTP layer and protective layers from the EL/FL layer, and programming a new set of patches on the EL/FL layer. The RE-TriLED arrays were operated at an alternating current (AC) frequency and voltage of 1 kHz and 175 V, respectively.

### SEM Characterization

Scanning electron microscopy (SEM) characterizations were performed with a JSM-7610F-Plus system at an accelerating voltage of 15 kV. The RE-TriLED was prepared as a sample for SEM using an ion-beam assisted cross-section polisher.

### Numerical Simulation

Numerical simulations of the electric field distribution in the RE-TriLED were performed using a commercial software (COMSOL Multiphysics 5.6). Parameters used in the COMSOL simulation are listed in Table S1. To simplify the calculation, the RTP and EL/FL layers were assumed to be composed of only PVA and PDMS, respectively. The protective layer (PVA or PEO ion gel layer) was omitted in the model.

### Characterization of Individual Optical Modes

UV irradiation tests were conducted using 254 and 365 nm UV lamps with a power of 4 W (Sankyo Denki). Time-resolved photoluminescence (TRPL) decay profiles of RTP, FL, and FLʹ emissions were obtained using a Fluorolog3 instrument (HORIBA) equipped with a 254 nm excitation light source at room temperature. The absorption spectra of RTP films, fluorescein, and rhodamine B were obtained using UV–Vis spectroscopy (V-650, JASCO). Fourier-transform infrared (FTIR) spectroscopy was conducted using a Vertex 70 system (Brunker). Impedance measurements were performed by precision LCR meter (E4980A, Keysight). The luminance, spectra, and CIE coordinates of the optical modes were obtained using a spectroradiometer (CS-2000, Konica Minolta). For driving the AC-EL, a function generator (33220A, Keysight) connected to an amplifier (623B, TREK) was used.

### Test of Thermal Stability

A test of thermal stability was performed using differential scanning calorimetry (DSC) and thermogravimetric analysis (TGA) with an SDT Q600 system. Measurement was performed during continuous heating with a heating rate of 5 K min^−1^.

## Results and Discussion

### Device Architecture and Optical Mode-Selective Operation of RE-TriLED

The device structure of a RE-TriLED is illustrated in Fig. 1a. An EL/FL layer (thickness of approximately 100 μm) consisting of ZnS:Cu particles (diameter, 25 ± 5 μm) embedded in polydimethylsiloxane (PDMS) was fabricated by spin coating a composite solution on two coplanar transparent indium tin oxide (ITO) electrodes separated with a gap of 1 mm on a glass substrate. The insulating PDMS matrix enables even distribution of the AC field and protects the display from breakdown [23]. An RTP layer of approximately 20 μm in thickness was subsequently prepared by spray coating a solution mixture of 5ʹ-m-terphenylboronic acid (DPP-BOH) and poly(vinyl alcohol) (PVA) on the EL/FL layer. Moreover, a protective layer of approximately 100 nm in thickness, composed of either PVA or poly(ethylene oxide) (PEO) ion gel with nonvolatile ionic liquid, 1-ethyl-3-methylimidazolium bis(trifluoromethylsulfonyl)imide (EMI-TFSI), was developed by spin coating on top of the RTP layer. In addition to the physical protection of the underlying RTP layer, the protective layer facilitates absorption and wetting of polar liquids or water into the RTP layer. Figure 1b shows a RE-TriLED fabricated on a 2.5 cm × 2.5 cm substrate ready for application of a polar liquid with a liquid-soaked brush. Successful fabrication of the RE-TriLED was confirmed by a cross-sectional view of the device using an ion-beam-assisted cross-section polisher, as shown in Fig. 1c.

The number of modes in the RE-TriLED can be controlled by combinations of external stimuli, such as UV light, AC field, and types of polar liquids, as shown in Fig. 1d. The mode-selective light emission of FL, RTP, and EL in a RE-TriLED is experimentally demonstrated in Fig. 1e. Initially, without a polar liquid on top of the protective layer acting as a floating electrode across the two coplanar ITO electrodes underneath, which is called a polar electrode bridge [22], the AC field applied between the coplanar electrodes did not lead to EL emission from the EL/FL layer because the AC field was mainly in-plane between the two ITO electrodes (Fig. 1e6, f (left) and Table S1) [24–26]. At this state, blue-green FL emission (mode 1) occurred from the EL/FL layer when the device was irradiated under UV light (wavelength of 254 nm), as shown in Fig. 1e4. When the UV was turned off, long-lasting greenish-blue RTP (mode 2) appeared, as presented in Fig. 1e5. Interestingly, 365 nm UV exposure did not trigger RTP emission, while the FL layer maintained its characteristic blue-green emission. This suggests that the RE-TriLED can be endowed with an encryption function in which the number of modes differs by the excitation wavelength (single-mode of FL or dual-mode of FL and RTP, as shown in Fig. 1e4–e6), which will be further discussed below.

When polar liquids were added on top of the protective layer of the RE-TriLED, the impedance of the RTP layer was substantially lowered (Note S1). The liquids including ethanol or ethylene glycol serve as a polar electrode bridge under the AC field between the coplanar ITO electrodes, and the dipoles of the liquids on top of the ITO electrodes are oppositely polarized [22]. In this situation, the AC field is then converted from in-plane to vertical, being concentrated at the EL/FL layer, and bluish-green EL emission (mode 3) occurred (Figs. 1e9 and S1). Because the AC field is not concentrated at the overlapping area between the EL/FL layer and interelectrode gap, light emission did not occur in this region (Fig. S1) [22]. Previous studies have established the AC-EL operation mechanism using a combination of device simulation models and experiments [22, 24–27]. It should be noted that because RTP emission still occurs when polar liquid is added to the RE-TriLED, our device can emit all three modes with the combination of AC field and UV exposure, as shown in Fig. 1e7–e9. When water was employed in the RE-TriLED, the device emitted characteristic EL from the EL/FL layer under an AC field because water again served as a polar electrode bridge (Fig. 1e3, f (right) and Table S1). Interestingly, however, when water was applied to the display, the RTP emission was deactivated (quenched), as shown in Fig. 1e2. At this stage, a single-mode of FL or EL can be achieved by the independent usage of a UV or AC field, and a dual mode when using both, as presented in Fig. 1e1–e3. Because a polar electrode bridge developed with either polar liquid or water can be erased by evaporation of the polar liquid with proper heat treatment, the system is reset to an initial stage, making our RE-TriLED rewritable, as schematically shown in the left of Fig. 1e.

Figure 1g shows the emission spectra of each mode, which correspond to the colors shown in Fig. 1e. RTP had a maximum emission wavelength at 475 nm with a quantum yield of 9.5% (Fig. S2b). Moreover, FL (full-width at half-maximum (FWHM), 99 nm) with a 47.8% quantum yield and EL (FWHM, 81 nm) both had their maximum at 500 nm (Fig. S2a). Therefore, our RE-TriLED enables various functions that could not be achieved via previous light-emitting optical encryption platforms, which meets the need for high-security information encryption (Fig. 1h and Table S2). Here, we define mode-selective operation as not only having independent control of each mode but also having interplays between the modes (EL and RTP for the RE-TriLED).

### Characteristics and Performance of Individual Optical Modes

**Fig. 2:**
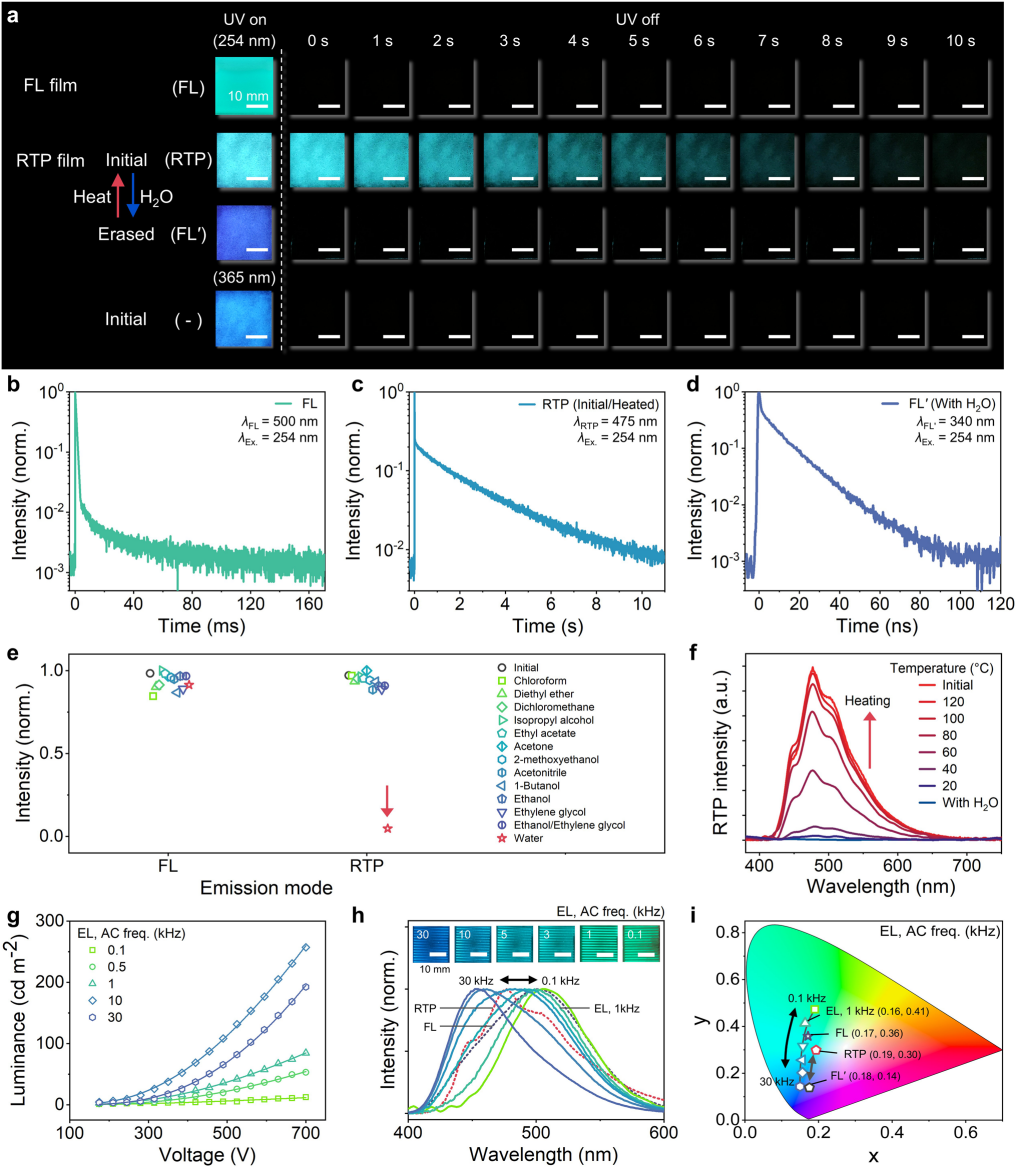
Characterization of individual emission modes. **a** Photographs of FL and RTP films under UV irradiation (254 or 365 nm) and after removal of UV lamp. **b**-**d** Time-resolved photoluminescence (TRPL) decay profiles of FL (**b**), RTP (**c**), and FLʹ (**d**) emissions. **e** FL and RTP intensity upon exposure to various liquids. **f** RTP spectra of an RTP film heated at different temperatures after exposure to H_2_O. **g**-**i** Luminance–voltage curves (**g**), normalized spectra (**h**), and CIE coordinates (**i**) of the EL emission as a function of AC frequency (polar-bridged by H_2_O) together with the ones from FL, RTP, and FLʹ emissions. The solid lines in **g** are a fit to an exponential function. The insets in **h** are photographs of EL emission under AC frequencies from 0.1 to 30 kHz

Prior to the investigation of encryptions utilizing the optical mode-selective properties of our RE-TriLED, we examined the characteristics and performance of each optical mode by using an FL film (ZnS:Cu/PDMS) and RTP film (DPP-BOH-PVA) each coated on a glass substrate. First, the photographs in Fig. 2a verify that an initial RTP of the RTP film was deactivated upon water treatment, and blue FLʹ emission appeared with a quantum yield of 10.1% (Figs. S2c and S3). On the other hand, the RTP film treated with either ethanol, ethylene glycol (EG), or a mixture of the two liquids still exhibited the characteristic RTP (Figs. S4 and S5 and Movie S1). As mentioned above, the RTP film did not show emission under 365 nm UV exposure (bottom of Fig. 2a), while the FL film maintained its maximum emission wavelength and color at both 365 and 254 nm excitation (Fig. S6). In the time-resolved photoluminescence decay profiles shown in Fig. 2b, blue-green FL from the EL/FL film showed a rapid decay with a lifetime of 0.17 ms. The RTP film before water treatment exhibited RTP with its lifetime reaching up to 1.85 s at 475 nm, whereas FLʹ with a lifetime of 11.2 ns was observed in the film with water (Fig. 2c, d, respectively).

To further examine the selective response of the RTP film and solvent stability of the FL film, the two films were exposed to a variety of liquids, as shown in Fig. 2e. As expected, quenching of the FL emission did not occur in all cases, while RTP was only deactivated upon water treatment. We note that this is one of the key features of the two films that enable mode-selective operation of the RE-TriLED. To confirm the RTP quenching mechanism of the RTP layer with water, various characterizations were conducted of the RTP films. UV–Vis absorption spectrum of the RTP film without water treatment showed an absorption peak at approximately 449 nm, which may be derived from the formation of B–O covalent bonds between DPP-BOH and PVA (Fig. S7a) [20]. The covalent bonds induce structural rigidification that hinders the thermal motion of DPP-BOH molecules which, in turn, reduces the nonradiative decay and quenching of the triplet [12, 21]. Moreover, the immobilized structure enhances the intersystem crossing from the singlet state to the triplet state of DPP-BOH, and RTP emission is facilitated from the DPP-BOH chromophores [21]. Meanwhile, the peak was rarely observed from a film treated with water. The UV–Vis spectroscopy results were corroborated by Fourier-transform infrared (FTIR) spectroscopy (Fig. S7b). The RTP film without water exhibited a noticeable peak at approximately 3329 cm^−1^, which could be ascribed to the associated hydroxyl group between adjacent DPP-BOH-PVA. When the film was fumed with water, the peak became blunt, and the peak position was shifted to approximately 3304 cm^−1^, which indicates that the water in the film could increase the degree of association of hydroxyl groups. Destruction of hydrogen bonds between the PVA chains in the RTP film occurs, and instead the PVA chains form hydrogen bonds with the water molecules [20]. This water-responsive property is facilitated by PVA, possibly due to the hygroscopic nature of the PVA, as shown for other PVA-based phosphorescent materials [21, 28, 29]. The deactivation of RTP did not occur when treated with isopropyl alcohol, 2-methoxyethanol, 1-butanol, ethanol, and ethylene glycol, molecules that could form hydrogen bonds (Fig. 2e). We speculate that this is because the hydrogen bond energy of these molecules are not strong enough to destruct the intermolecular hydrogen bond interaction between the adjacent PVA chains.

To evaluate the recovery of RTP emission of a water-treated RTP film upon evaporation of water by heat, we measured how the RTP intensity changed when heating a water-treated RTP film on a hotplate, and the results are shown in Fig. 2f (Fig. S8). Initially, the water-treated film showed an almost complete absence of RTP emission at 475 nm. Then, the RTP intensity gradually increased when exposed to a higher temperature, and upon reaching 100 °C, the RTP intensity plateaued. Further temperature increases led to minor enhancement of the intensity. Around this point, the film was nearly devoid of moisture, but for thorough moisture elimination within the device, we proceeded with RTP recovery tests by heating the RE-TriLED at 120 °C for 5 min. It should be noted that the RTP film was rarely decomposed up to 120 °C using thermogravimetric analysis (TGA) and differential scanning calorimetry (DSC) (Fig. S9). The stability of the RTP film was also confirmed by only showing a slight decrease in its intensity after 2 days under ambient conditions (relative humidity [RH] of 30%–50% and 20 °C) (Fig. S10). The quenching speed of the RTP can be regulated by different RH conditions, where the RTP is maintained for RH under 60% while it showed an abrupt decrease over 80% (Fig. S11). Therefore, this characteristic could be used for time-dependent encryption of the RE-TriLED, where information encrypted in the RTP layer disappears after a certain amount of time [9]. The RTP film can easily recover back to its initial state even after being left for a long period of time or being exposed to high RH conditions. For a wider implementation of our RE-TriLED, encapsulating the display with nonpolar PDMS would mitigate the quenching of the RTP emission, enabling the encrypted information to be retained even at RH over 80% (see Fig. 6a, right).

EL performance of the RE-TriLED was examined with a polar electrode bridge developed with water, and the results are shown in Fig. 2g–i. The RE-TriLED with a water droplet on top of the protective layer exhibited a progressive enhancement in luminance in response to increasing AC voltage and frequency but showed a decline in luminance when the frequency exceeded 10 kHz, consistent with the results shown by previous AC-EL displays (Fig. 2g) [30]. A similar EL performance was observed from our RE-TriLED with an ethanol or EG polar electrode bridge, as expected (Fig. S12). In addition, a distinctive emission color transition of the EL was observed, with the emission color migrating from green to blue in line with the AC frequency increasing from 0.1 to 30 kHz, as shown in Fig. 2h [30–32]. In ZnS:Cu, two activator centers exist which correspond to blue and green EL emission, respectively. Increasing the AC frequency leads to the population of electrons in the green activator center to saturate, causing the spectral shift [33]. The wavelength at the maximum EL intensity was approximately 506 nm at 0.1 kHz, and it became approximately 454 nm at 30 kHz. Emission color adjustability was confirmed by photographs and CIE coordinates as a function of frequency shown in Fig. 2h inset and Fig. 2i, respectively. Thus, by operating a RE-TriLED at an AC frequency of 1 kHz (voltage of 175 V), the RE-TriLED is able to exhibit three emissions of FL, RTP, and EL with distinct CIE coordinates, as shown in Fig. 2i. The emissions of a RE-TriLED being three distinct colors are beneficial for developing a mode-selective encryption display, as discussed below.

### Mode-Selective RE-TriLED with Reversible RTP

**Fig. 3:**
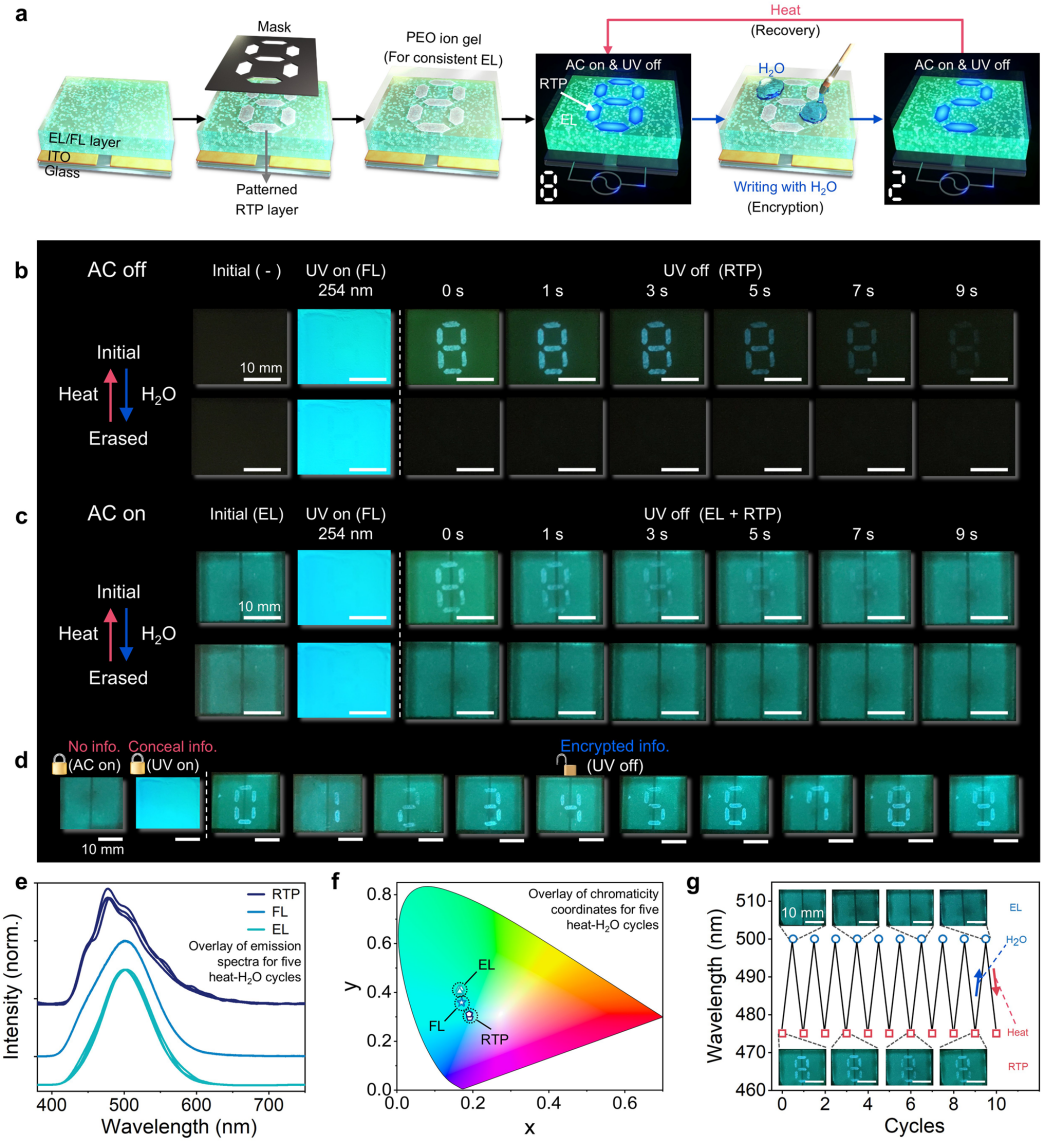
Mode-selective RE-TriLED with reversible RTP. **a** Schematic illustration of fabricating a RE-TriLED with a patterned RTP layer, and encryption process using H_2_O and heat exposure. **b**, **c** Photographs of a RE-TriLED under UV irradiation and after removal of UV lamp without (**b**) and with (**c**) AC field. The RE-TriLED is also exposed to either H_2_O or heat for reversible RTP emission. **d** Photographs of a RE-TriLED showing an encrypted number from zero to nine by treating H_2_O on selective areas of the patterned RTP layer. **e**, **f** Overlay of normalized spectra (**e**) and CIE coordinates (**f**) of RTP, FL, and EL emissions for five heat-H_2_O cycles. **g** RTP and EL emission wavelengths of a RE-TriLED upon repetitive H_2_O and heat exposure (inset shows photographs of the RE-TriLED during the repeatability test)

Based on the RTP deactivation by water and its reappearance by subsequent heating process, the RTP information programmed in a RE-TriLED was reversible without interfering the other two modes, as shown in Fig. 3. The reversibility of RTP in a RE-TriLED was examined by the process shown in Fig. 3a. To write RTP information on the device, an RTP layer was patterned via spray coating onto an EL/FL layer, followed by spin coating a PEO ion gel layer on top. One digit, the number “8”, was programmed with the patterned RTP layer. The PEO ion gel layer with EMI-TFSI served as a polar electrode bridge for constant EL emission regardless of the presence and position of water on the RE-TriLED (Fig. S13) [25]. We compared how the EL performance and impedance change with the ratio between the polymer and ionic liquid consisting of ion gel (Fig. S14). Both impedance and EL luminance showed a minor change when the ratio of PEO to ionic liquid was higher than 1:3. Thus, the 1:3 ratio was implemented together with an AC frequency and voltage of 1 kHz and 175 V, respectively. In this step, the “8” could be seen when UV was turned off. Notably, the whole device exhibited EL emission under an AC field in addition to FL emission upon UV exposure. The RTP information of digit “2” appears when some parts of the digit “8” were treated with water, as shown in the last scheme of Fig. 3a. The entire device still emits EL and FL under an AC field and UV exposure, respectively. By evaporating water treated on the specific parts of the digit “8”, the digit “8” reappeared when UV was turned off. The RTP pattern manipulation by water and subsequent heating occurs reversibly, enabling reversible RTP encryption with our RE-TriLED.

When the RE-TriLED with the patterned “8” RTP layer was exposed to 254 nm UV, the entire device emitted FL, as shown in Fig. 3b. When UV was turned off, the patterned RTP information of “8” was apparent, and the intensity of the RTP gradually decreased with time, as shown in the series of photographs at the top of Fig. 3b. After water treatment on the device, no RTP was observed when UV was turned off, as shown in the series of raw photographs at the bottom of Fig. 3b. As described previously, when the water was evaporated by heat, the RTP with the patterned “8” reappeared in UV-off mode.

The display showed characteristic EL emission from the entire area without any information when an AC field was applied to the RE-TriLED, as shown in Fig. 3c. In addition, the 254 nm UV exposure on the RE-TriLED resulted in FL, followed by RTP emission with the patterned digit “8” when UV was turned off, as shown in the series of raw photographs at the top of Fig. 3c. The operating AC voltage was carefully regulated to a level where EL emission did not hide the FL emission, even when an AC field and UV were applied at the same time (Fig. S15a, b). Because the RE-TriLED was operated at an AC frequency that produced bluish-green EL emission, the information of the RTP emission was still visible when an AC field was applied, as shown in Figs. 2i and 3c (Fig. S15c, d). Specifically, the EL and RTP emissions maintained their wavelengths at approximately 500 and 475 nm, respectively (Fig. 1g). As shown in the series of raw photographs at the bottom of Fig. 3c, applying water on the entire area of the RE-TriLED caused total elimination of the RTP emission, whereas both EL and FL emissions were unaffected by water under AC field and UV exposure, respectively (Figs. 2e and S13). The RTP emission was restored when heating the device enough to evaporate the water applied to the RE-TriLED.

As schematically illustrated in Fig. 3a, the patterned RTPs with different digit numbers from “0” to “9” could be written by selectively treating water on the parts of the initially written “8”, and the results are shown in Fig. 3d (Movie S2). The encrypted number produced by the patterned RTP layer, followed by the selective treatment with water, was only visible when a UV lamp of a particular wavelength (254 nm) was turned off. When the RE-TriLED with the encrypted RTP digit information was continuously exposed to a UV lamp, exposed to a 365 nm UV lamp, or only given an AC field, no information appeared. As mentioned above, the encrypted digit could gradually disappear with time when exposed under high RH conditions, enabling time-dependent encryption. In addition, the encrypted number could be reset to the initial digit “8” by heating the device, and a new encrypted digit could be written by the selective treatment with water.

To evaluate the stability of the RE-TriLED, we examined the emission spectra and CIE coordinates of the three emission modes during the heat-H_2_O cycle, as shown in Fig. 3e, f, respectively. All three modes showed stable operation without significant alternation in the intensity and color. Through 10 cycles of quenching and restoring the RTP emission of the RTP layer, we confirmed the reversibility of RTP, as shown in Fig. 3g. The reliable operation of the device was also attributed to the thermally stable PEO ion gel layer on the patterned RTP, which served as not only a water protective layer but also as a polar electrode bridge for the EL emission.

### RE-TriLED with Full Visible RTP

**Fig. 4:**
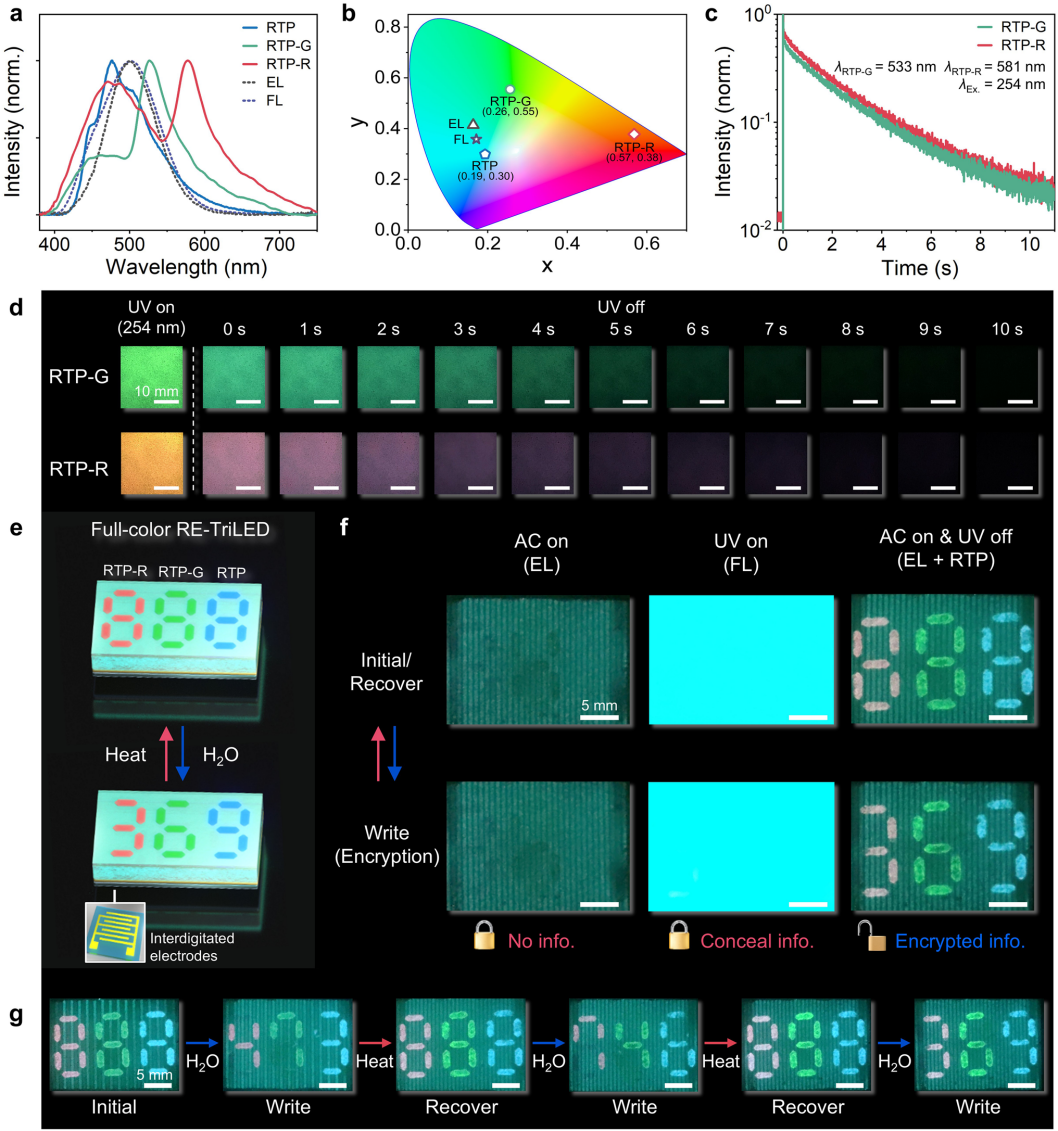
RE-TriLED with full visible RTP. **a**, **b** Normalized emission spectra (**a**) and CIE coordinates (**b**) of RTP, fluorescein-doped RTP (RTP-G), and rhodamine B-doped RTP (RTP-R) films together with the ones from EL and FL emissions. **c** TRPL decay profiles of RTP-G and RTP-R films. **d** Photographs of RTP-G and RTP-R films under UV irradiation and after removal of UV lamp. **e**, **f** Schematic illustration of a full-color RE-TriLED showing encrypted numbers by reversible RTP emission (**e**) and photographs of the full-color RE-TriLED exposed to UV and/or AC field (**f**). **g** Photographs of a full-color RE-TriLED showing different numbers encrypted upon repetitive heat-H_2_O exposure

To facilitate RTP emission in the full visible wavelength range, we added fluorescent dyes, specifically, fluorescein and rhodamine B, to the RTP films (DPP-BOH-PVA). The methodology was rooted in the research conducted by George et al. [34], who realized triplet-to-singlet Förster-resonance energy transfer by using phosphors with extended lifetimes as energy donors and fluorescent dyes as energy recipients [35]. In our system, DPP-BOH-PVA acts as an energy donor, while fluorescein and rhodamine B serve as energy acceptors for the green and red spectra, respectively (Fig. S16). For the afterglow spectrum of a fluorescein-doped RTP film (RTP-G), the primary emission was observed at its maximum peak located at 533 nm arising from the fluorescein, as shown in Fig. 4a. A weak emission peak additionally appeared at 475 nm, mainly resulting from DPP-BOH-PVA, which suggests an efficient energy transfer from DPP-BOH-PVA to fluorescein. In the rhodamine B-doped RTP film (RTP-R), two emission peaks with similar intensity were apparent at wavelength values with the maximum emission of approximately 581 and 475 nm arising from rhodamine B and DPP-BOH-PVA, respectively (Fig. 4a). The similar intensity values of the two peaks were due to the small spectral overlap between the absorption spectrum of rhodamine B and the phosphorescence spectrum of DPP-BOH-PVA (Fig. S17). The wavelength-controlled RTP emission was obtained in the RTP-G and RTP-R, as confirmed by the CIE coordinates of Fig. 4b. Furthermore, time-resolved photoluminescence decay profiles of the RTP-G and RTP-R films showed lifetimes of 1.60 s (at 533 nm) and 1.90 s (at 581 nm), respectively, as shown in Fig. 4c. Their long afterglows were visually detected for approximately 10 s, like the original RTP film, as shown in the series of photographs in Fig. 4d.

Both RTP-G and RTP-R were also reversible when employed in RE-TriLEDs, and the results are shown in Fig. S18. As the emission spectrum of either RTP-G or RTP-R did not completely overlap with the EL emission showing bluish green, the digit numbers fabricated by spray coating either RTP-G or RTP-R were clearly distinguished from the EL emission when both UV and an AC field were applied (Fig. 4a, b and S18). Leveraging the wavelength-tunable properties of the RTP films, we constructed a reversible RE-TriLED with full-color, multi-digit number encryption capability, as schematically shown in Fig. 4e. The three RTP, RTP-G, and RTP-R patterns with the digit “8” were spray coated on a single EL/FL layer using the patterned masks, as previously described in Fig. 3a. A PEO ion gel layer was subsequently spin coated on top of the patterned RTP layers, ensuring reliable EL emission from the whole device (Fig. S13). As shown in the top left photograph of Fig. 4f, applying an AC field to the display enabled EL emission from the entire area without showing patterned (encrypted) information. UV irradiation caused the FL emission to conceal the encrypted information (top middle photograph of Fig. 4f). Removal of the UV lamp led to RTP emission from the “888” pattern with different colors (Fig. S19). When UV was turned off while the AC field was on, vivid RTP emission was apparent on the background EL emission, as shown in the top right photograph of Fig. 4f.

Next, water was treated on the selected areas of the previously patterned RTP digits to deactivate the RTP emission on the treated areas, as schematically shown in Fig. 4e. For instance, the two parts of the RTP-R digit “8” were treated with water, giving rise to an expected RTP emission with the digit “3”. Likely, water was further treated on the RTP-G digit “8” and the RTP digit “8” to produce the resulting RTP emission with the digits of “6” and “9” from the RTP-G and RTP, respectively. The RE-TriLED with water treatment also exhibited the characteristic EL upon AC field, as shown in the bottom left photograph of Fig. 4f. In UV-on mode, only FL emission was shown, identical to the case without water treatment (bottom middle photograph of Fig. 4f). When UV was turned off while the AC field was on, vivid RTP emission from the “369” pattern with different colors was apparent on the background EL emission, as shown in the bottom right photograph of Fig. 4f (Movie S3). The reversibility was confirmed by sequential encryption of digits “473”, “746”, and “369” on the full-color RE-TriLED, as shown in Fig. 4g.

### Rewritable RE-TriLED with EL and RTP Interplay

**Fig. 5:**
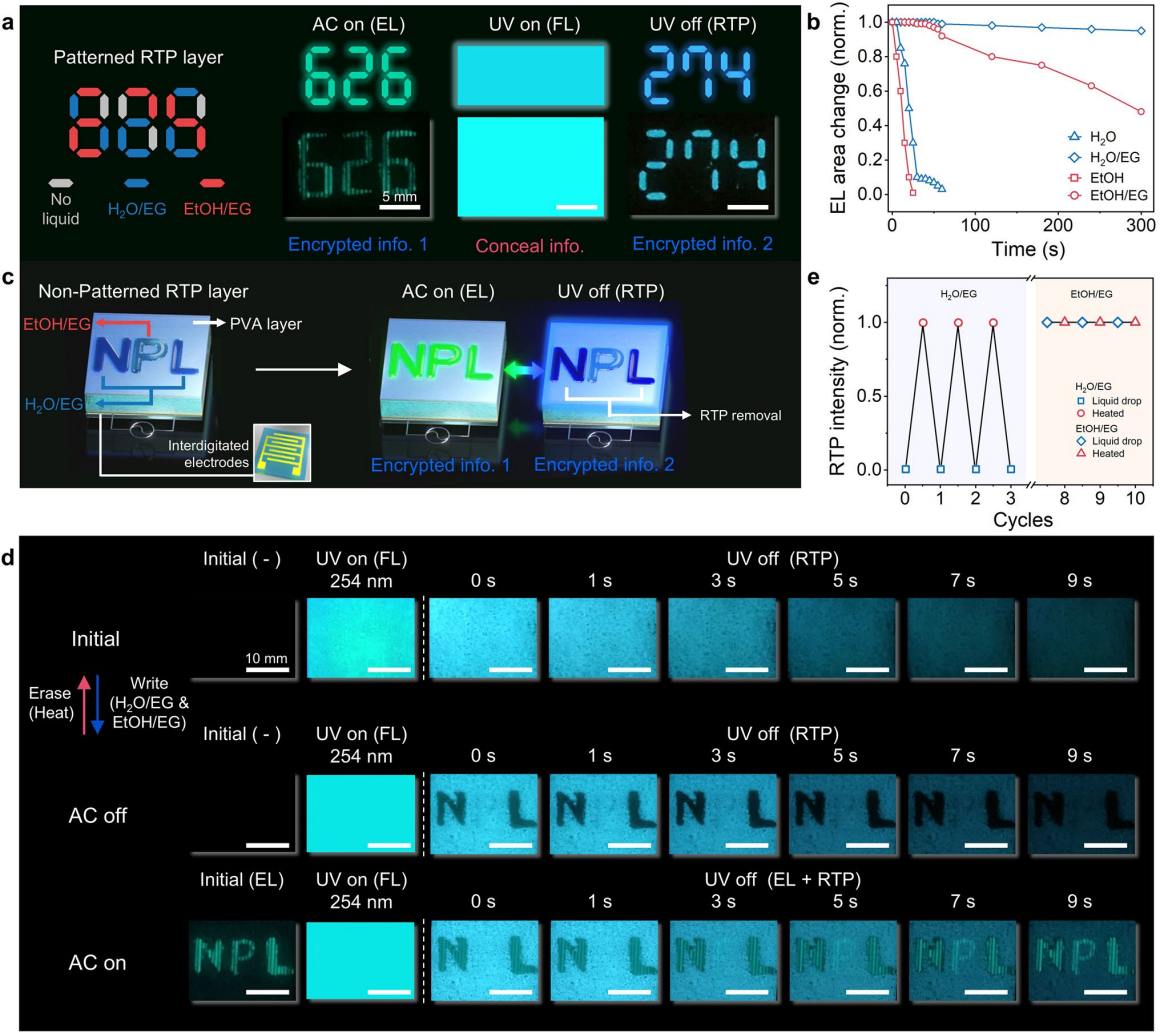
Rewritable RE-TriLED with EL and RTP interplay. **a** Multilevel encryption of RE-TriLED with a patterned RTP layer using H_2_O/ethylene glycol (EG) and EtOH/EG mixture. **b** Normalized EL area change upon exposure to H_2_O, EtOH, or their mixtures with EG. **c** Schematic illustration of writing letters on a nonpatterned RE-TriLED. **d** Photographs of the RE-TriLED illustrated in **c**, exposed to UV and/or AC field. **e** Normalized RTP intensity upon repetitive exposure to H_2_O/EG or EtOH/EG mixture

As mentioned above, interplay between the modes enables complex scenarios where independent information can be encrypted on each mode. To enable this, additional writing processes were employed to the EL/FL layer, based on the type of polar liquids, as schematically shown in Fig. 5a. In the EL writing processes, a PEO ion gel layer (Figs. 2–4), was not employed (Fig. S13). Instead, a PVA layer was prepared on a patterned RTP layer treated with water and ethanol inks mixed with EG. First, the digits “888” were patterned with an RTP layer via spray coating. The selected areas of the “888” were treated with either water/EG or ethanol/EG mixture, as illustrated in the leftmost scheme of Fig. 5a. As evidenced in Fig. 5b, the polar liquid retainability can be regulated by the employment of EG, which enables time-dependent encryption of the EL information [10]. A RE-TriLED with either water or ethanol serving as a polar electrode bridge was turned on with EL upon AC field application, but the EL emission rapidly degraded with time due to the fast evaporation of the liquid in air. The EL of the device became substantially more persistent when EG was mixed with either water or ethanol due to the high boiling point of EG, giving rise to an EL emission over 300 s (Figs. S20 and S21).

Because both water/EG and ethanol/EG can develop a polar electrode bridge in a RE-TriLED, enabling EL emission under an AC field, the digits of “626” written with both liquid mixtures appear under the AC field, as shown in the left scheme and photograph of Fig. 5a (Fig. S22). When 254 nm UV was exposed to the RE-TriLED, the entire device emitted FL from the EL/FL layer, as shown on the middle scheme and photograph of Fig. 5a (Fig. S22). In this situation, EL and RTP were also emitted from the patterned digits, but due to the high intensity of FL, both information were hidden. When UV was turned off, the RTP information was seen. As RTP from the regions treated with water/EG mixture was deactivated, the patterns except the water-treated regions emitted RTP in UV-off mode. In our RE-TriLED, the digits “274” appeared in RTP, as shown in the right scheme and photograph of Fig. 5a (Fig. S22 and Movie S4). We note that the situation can be further modified such that another information is encrypted in the FL emission (third encrypted information) by patterning the EL/FL layer, which acts as a background [15], but such scenarios were not considered here.

A direct-writing encryption of a RE-TriLED with the independent encryption of EL and RTP was also developed, as schematically shown in Fig. 5c. First, a RE-TriLED was fabricated with a nonpatterned RTP layer covered with a polar liquid-absorbable PVA protective layer. In this example, the letters “N” and “L” were directly written on an RTP layer with a water/EG mixture ink, while the letter “P” was written with the ethanol/EG mixture ink, as shown in the left scheme of Fig. 5c. When an AC field was applied to the device, the letters “N”, “P”, and “L” were recognized with the AC-EL due to the development of polar electrode bridges with both the water/EG and ethanol/EG inks, as shown in the middle scheme and photograph in Fig. 5c, d, respectively. When 254 nm UV was applied, the entire RE-TriLED device emitted FL from the EL/FL layer (Fig. 5d). Again, the EL and RTP were much weaker than the FL, making the FL emission dominant in the device. The characteristic RTP was apparent when the UV was turned off. In our encrypted RE-TriLED, the letters “N” and “L”, which had been written with the water/EG mixture ink (the RTP deactivation agent), became dark, while the rest of the device emitted the RTP, as shown in the right scheme and photographs of Fig. 5c, d, respectively. In this encryption display, the first layer of a password was set by the two inks, allowing password decryption by the AC field. The second layer of a password was obtained in RTP mode by the UV on and off process. When an AC field was immediately applied to a RE-TriLED after UV was turned off, the letters of “N”, “P”, and “L” reappeared in EL mode on the RTP background, as shown in the series of photographs in Fig. 5d (Movie S4). Our RE-TriLED with the independent EL and RTP encryption was again rewritable because the information written by either water/EG or ethanol/EG mixture inks was readily erased by heat, followed by re-programming new information with the two inks, as confirmed by the results of rewritable RTP of a RE-TriLED with the two inks (Figs. 5e and S23).

### Rewritable Multilevel Encryption of RE-TriLED Array

**Fig. 6:**
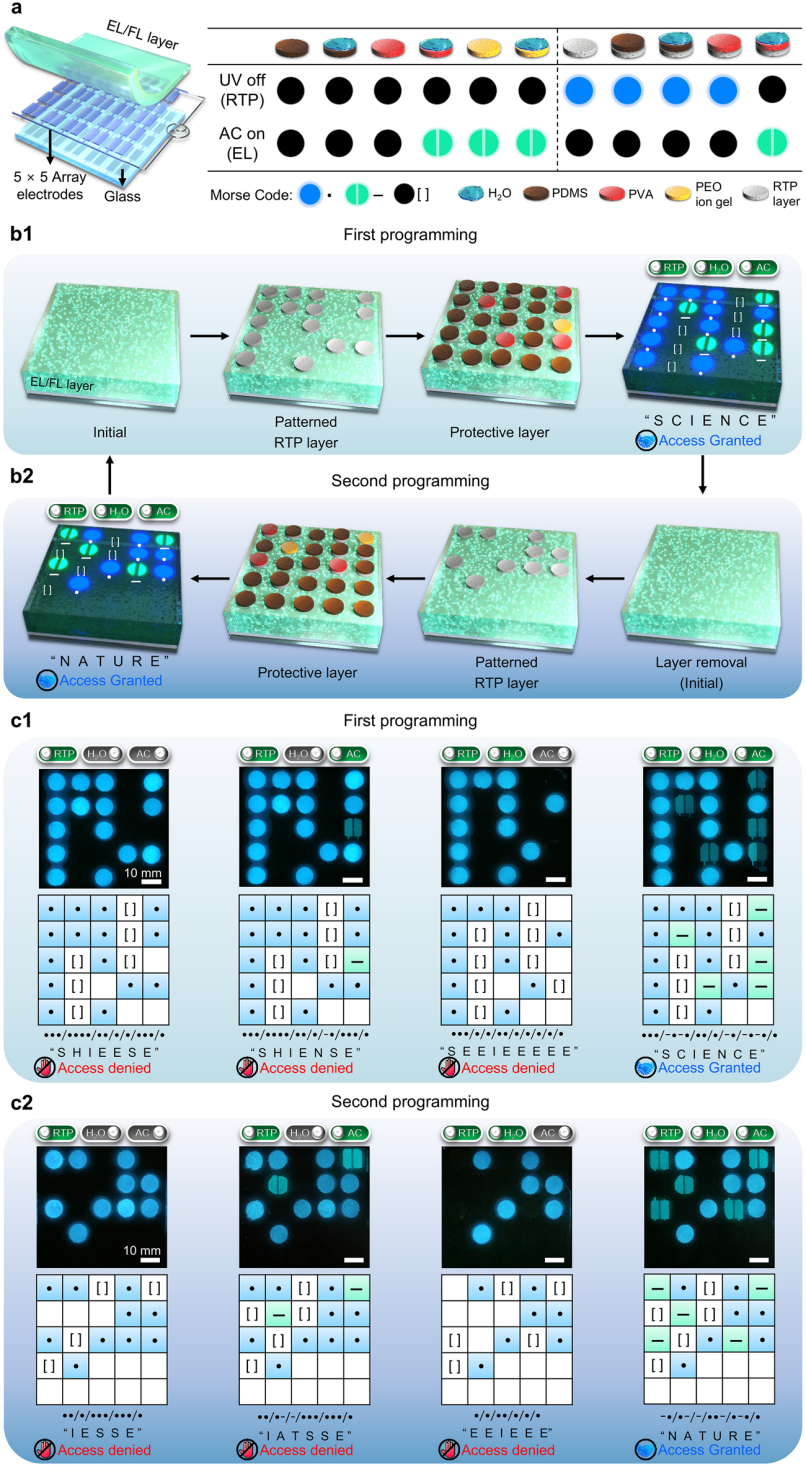
Rewritable multilevel encryption of RE-TriLED array. **a** Left: exploded view layout of pixelated RE-TriLED array. Right: possible combinations of layers and their response to UV and AC field. **b** Schematic illustration of fabricating RE-TriLED arrays programmed with Morse code (**b1**) and its rewritability by removal of RTP and protective layer (**b2**). Real information is shown only when the RE-TriLED is exposed to all three stimuli. **c** Cases for the rewritable RE-TriLED array. **c1** Photographs and schematic illustrations of the RE-TriLED array illustrated in **b1**, where the display is exposed to UV, UV and AC, UV and H_2_O, and all together. **c2** Photographs and schematic illustrations of the RE-TriLED array after its second programming, where the display is exposed to UV, UV and AC, UV and H_2_O, and all together

Capitalizing on the rewritable and mode-selective properties of a RE-TriLED with the independent encryption of EL and RTP, we developed a rewritable high-information-security encryption display consisting of 5 × 5 array of RE-TriLEDs, as shown in Fig. 6. Morse code, a communication method used to encode text characters through standardized sequences of two different signal durations—dots and dashes or dits and dahs—has been in use for a long time. International Morse code can encode a small set of procedural signals, punctuation, Arabic numerals, and 26 basic Latin letters from A to Z, enabling the design of a Morse code system through human perceptible information, such as sound, vibration, or visible light [11, 31]. A novel Morse code-based encryption display was successfully developed with the array of RE-TriLEDs, as schematically shown in Fig. 6a. The display was fabricated by depositing 5 × 5 array of two parallel electrodes connected to an AC power source on a glass substrate. An AC field from the power source was applied to all arrays of the electrodes at the same time. An EL/FL layer was deposited on the arrays of the electrodes (Fig. 6a, left). To manipulate the individual pixels of the display, we fabricated four different circular patches of 7 mm in diameter of RTP layer, nonpolar PDMS (polar liquid not absorbable), polar liquid-absorbable PVA, and PEO ion gel with EMI-TFSI ionic liquid, as schematically shown in Fig. 6a, right. In our setup, the encoded information is presented as Morse code signals, where the RTP emissions represent “dots”, and the EL emissions serve as “dashes”. Areas devoid of either emission are referred to as cells or denoted by “[]”. Reading Morse code signals in our system involves recognizing sequences of consecutive dots or dashes, with the principle that a cell, represented by “[]”, signifies a transition to the next letter.

First, the RTP patches were placed on the EL/FL layer, followed by adding three different patches of PDMS, PEO ion gel, and PVA as a protective layer, as schematically shown in Fig. 6b1. Multilevel encryption is achieved from the RE-TriLED array in which the display could show multiple information that are wrong when one or two stimuli is applied. The real password information of “SCIENCE” will only show when water is applied to the display together with AC-on and UV-on–off process, which will be further discussed below. To encode a new information, all four different patches placed on the 5 × 5 array of devices were readily detached from the EL/FL layer, and a new set of patches were placed on the EL/FL layer, as schematically shown in Fig. 6b2. Similarly, the information of “NATURE” can only be achieved when all stimuli are appropriately applied to the RE-TriLED array.

The photographs and schematic illustrations of Fig. 6c1 show cases that could occur from the RE-TriLED array in Fig. 6b1. When 254 nm UV was turned off, the pixels with the RTP patches emitted RTP, as shown in the leftmost of Fig. 6c1. From the programmed RTP emission in our display, the first four characters S, H, I, and E were read as “•••” (S), “••••” (H), “••” (I), and “•” (E), respectively, giving rise to an unfamiliar set of characters “SHIEESE”. When an AC field was immediately applied to the array of RE-TriLEDs after UV was turned off, EL emission was added from pixel (3,5), where PEO ion gel was used, as shown in the second photograph and scheme of Fig. 6c1. As the pixels with EL were assigned as dashes in Morse code, the Morse code information changed to “SHIENSE”. To add encrypting information, selective treatment of the PVA patched pixels was performed with water. When 254 nm UV was turned off, the pixels previously patched with RTP covered with PDMS emitted RTP, whereas the RTP patched pixels of (1,5), (2,2), and (4,5), additionally covered with PVA patches followed by water treatment, did not show RTP due to the water-induced deactivation of RTP on the pixels, as shown in the third photograph and scheme of Fig. 6c1. With the RTP-emitting arrays of the device, the Morse code information of “SEEIEEEEE” was obtained. Additional encryption information was achieved when an AC field was applied to the array of RE-TriLEDs after water treatment, as shown in the rightmost of Fig. 6c1. In this situation, the pixels with polar electrode bridges emitted EL under an AC field in addition to the RTP-emitting pixels. The pixels with the water-treated PVA patches (1,5), (2,2), (4,3), and (4,5) emitted EL. In addition, the pixel (3,5) with a PEO ion gel patch emitted EL due to the polar electrode bridge developed with the polar ionic liquid of EMI-TFSI in the patch. The real password information of “SCIENCE” was finally achieved.

After programming a new information for the RE-TriLED array (second programming), the pixels with the RTP patches emitted RTP when 254 nm UV was turned off, as shown in the photograph and scheme of Fig. 6c2. The Morse code information of “IESSE” was obtained from the RTP emissions on the array of devices, based on the reading procedure described previously. Next, when UV was turned off while the AC field was on, EL information from pixels (1,5) and (2,2) with PEO ion gel patches were added, as shown in the second photograph and scheme of Fig. 6c2. Together with the RTP information, the Morse code information of “IATSSE” was obtained. Water treatment of pixels (1,1), (3,1), and (3,4) with the PVA patches placed on the RTP patches deactivated the RTP of the pixel when UV was turned off. The RTP emission pattern from the array of the devices in UV-off mode was obtained such that the three pixels with the PVA patches treated with water were devoid of RTP emission. The resulting RTP emission pattern shown in the third photograph and scheme of Fig. 6c2 resulted in Morse code information “EEIEEE”. True Morse code information was finally obtained from the optical pattern with RTP and EL emission when an AC field was applied to the arrays with the various patches, as schematically shown in the rightmost of Fig. 6c2. From the RTP and EL emission from the selected pixels, the Morse code information of “NATURE” was achieved. The rewritable principles of our RE-TriLED with independent control of RTP and EL under UV-off and AC-on modes, respectively, offer a simple but robust route for developing a novel cost-effective high-level encryption display.

## Conclusions

This study demonstrated a rewritable mode-selective light-emitting display based on three light emissions of FL, RTP, and EL. A single-device display with a four-layer architecture exhibited mode-selective operations when manipulating the polar electrode bridge, which served as a switch for EL in the display, with the choice of water and polar liquids that deactivated and activated full visible color RTP, respectively. With the rewritable capability of our triple-mode display accomplished with a simple heat treatment, a rewritable optical encryption display with multi-security levels was developed in which real Morse code information encoded by RTP was decoded only when properly matched with that of the FL and EL. Our single-device triple-mode light emission platform underscores the considerable potential of emerging visual-encryption electronics, marking a stride forward in secure data storage and communication.

## Supplementary Information

Below is the link to the electronic supplementary material.

Supplementary file 1 (MP4 2900 KB)

Supplementary file 2 (MP4 1334 KB)

Supplementary file 3 (MP4 2018 KB)

Supplementary file 4 (MP4 2630 KB)

Supplementary file 5 (DOCX 13031 KB)
