# Supplementary material for: Rewritable Triple-Mode Light-Emitting Display
Source: Nanomicro Lett. 2025 Mar 13;17:183. doi: 10.1007/s40820-025-01686-4 (PMC11903999; doi:10.1007/s40820-025-01686-4)
Supplement: Supplementary file 5 — (DOCX 13031 KB) [file 40820_2025_1686_MOESM5_ESM.docx]

Supporting Information for

**Rewritable Triple-Mode Light-Emitting Display**

Seokyeong Lee^1,#^, Jong Woong Park^1,#^, Jihye Jang^1^, Jin Woo Oh^1^, Gwanho Kim^1^, Jioh Yoo^1^, Jong Gun Jung^1^, Hyowon Han^1^, Wei Jiang^1^, Chang Eun Lee^1^, Jungwon Yoon^1^, Kaiying Zhao^1^, and Cheolmin Park^1, 2, *^

^1^Department of Materials Science and Engineering, Yonsei University, 50 Yonsei-ro, Seodaemun-gu, Seoul 03722, Republic of Korea

^2^Post-Silicon Semiconductor Institute, Korea Institute of Science and Technology, 02792, Republic of Korea

^#^Seokyeong Lee and Jong Woong Park contributed equally to this work.

*Corresponding author. E-mail: [cmpark@yonsei.ac.kr](mailto:cmpark@yonsei.ac.kr) (Cheolmin Park)

**Note S1 Parameters for adjusting the EL performance**

Ideally, our RE-TriLEDs are equivalent to coplanar plate capacitors with a capacitance (*C*) proportional to the averaged relative permittivity (*ε_avg_*) of the layers stacked on the electrodes.

$C\propto\varepsilon_{avg}$ (S1)

The modulus of the impedance (|*Z*|) is inversely proportional to *C* such that

$\left| Z \right| \propto\left| \frac{1}{C} \right| \propto\left| \frac{1}{\varepsilon_{avg}} \right|$ (S2)

Thus, when polar liquids are added and absorbed into the RTP layer of the RE-TriLEDs, *ε_avg_* is increased and |*Z*| is reduced, causing the electric field distribution to convert from in-plane to vertical. The relative permittivity of water (H_2_O) is higher compared to ethanol (EtOH) or ethylene glycol (EG) (Table S1), which leads to a higher reduction of |*Z*| and a higher luminance (Figs. S12 and S21).

AC-EL devices involving inorganic phosphors are based on hot electron excitation mechanism [S1], and their EL performance is mainly determined by the voltage input. At fixed temperature and AC frequency, the relationship between the luminance (*L*) and input voltage (*V*) can be expressed through the following equation [S[1](#r1), S[2](#r2)]:

$L = aexp(-\frac{b}{V^{1/2}})$ (S3)

where *a* and *b* are constants determined by factors such as particle size, orientation between the particle and applied electric field, concentration of the particles in the dielectric layer, dielectric constant of the dielectric layer, and device thickness. The model fitted well with our experimental results, as shown in the solid lines in Fig. 2g.

**Supplementary Table and Figures**

**Table S1** Parameters used in numerical simulations

| Layer (Material) | | Parameters | | | |
| --- | --- | --- | --- | --- | --- |
|  |  | Electrical conductivity (S m^-1^) [S3, S4, S6] | Relative permittivity [S3, S5, S7] | Width  (mm) | Height  (mm) |
| RTP layer (PVA) | | 1×10^-8^ | 8.0 | 11 | 0.02 |
| EL/FL layer (PDMS) | | 3.1×10^-13^ | 2.7 | 11 | 0.1 |
| Electrode (ITO) | | 1.3×10^4^ | 1×10^10^ (Infinite) | 5 | 0.05 |
| Air | | 0.0 | 1.0 | 40 | 40 |
| Polar liquid | H_2_O | 2.0 | 80 | 11 | 0.5 |
|  | EtOH | 5.5×10^-5^ | 24.3 |  |  |
|  | EG | 1.1×10^-4^ | 37.7 |  |  |

**Table S2** Comparison of multi-light-emitting platforms

| Type | Material | Number of modes (Type) | Mode-selectively operable | Stimuli-responsive (Stimuli) | Rewritable | Time-dependent encryption | Refs |
| --- | --- | --- | --- | --- | --- | --- | --- |
| AC-EL | ZnS:Cu and  DPP-BOH-PVA | 3  (FL, RTP, EL) | O | O (Polar liquids, Humidity) | O | O | This  work **^c^** |
|  | ZnS:Cu | 2 (FL, EL) | X | O (Low-*Z* mater.) ^a^ | X | X | [S[8](#r8)] **^c^** |
|  | ZnS:Cu | 2 (FL, EL) | X | O (Low-*Z* mater.) | X | X | [S[9](#r9)] |
|  | ZnS:Cu | 2 (FL, EL) | X | X | X | X | [S[10](#r10)] |
| FL and/or RTP | PDA organogel | 3 **^b^**  (SC, FL, Hol) | X | O (ACN, Humidity) | X | X | [S[11](#r11)] **^c^** |
|  | PS-*b*-P2VP and Perovskites | 2 (SC, FL) | X | O (EtOH, NH_4_OH) | O | O | [S[12](#r12)] |
|  | DPP-BOH-PVA | 2 (FL, RTP) | X | O (Humidity) | X | O | [S[13](#r13)] **^c^** |
|  | Arylboronic acids and PVA | 2 (FL, RTP) | X | O (Humidity) | X | O | [S[14](#r14)] |
|  | PVA-100-P2-1 | 2 (FL, RTP) | X | O (Heat) | X | O | [S[15](#r15)] |
|  | o-BFT | 2( FL, RTP) | X | O (Light) | X | X | [S[16](#r16)] |
|  | (ETPP)_2_ZrCl_6_ | 2 (FL, RTP) | X | X | X | O | [S[17](#r17)] |
|  | Pb-based MOF and Perovskites | 2 (FL, RTP) | X | X | X | X | [S[18](#r18)] |
|  | PY16, PY13 | 1 (FL) | X | O (DMSO, Humidity) | X | O | [S[19](#r19)] |
|  | [MnCl_4_]^2-^[etpp]^+^_2_ | 1 (RTP) | X | O (Humidity) | O | X | [S[20](#r20)] |
|  | CT5-0 | 1 (RTP) | X | O (DMSO) | O | X | [S[21](#r21)] |

**a** *Z* = Impedance

**b** SC = Structural color, Hol = Bright-field hologram

**c** Presented as representative works in Fig. 1h


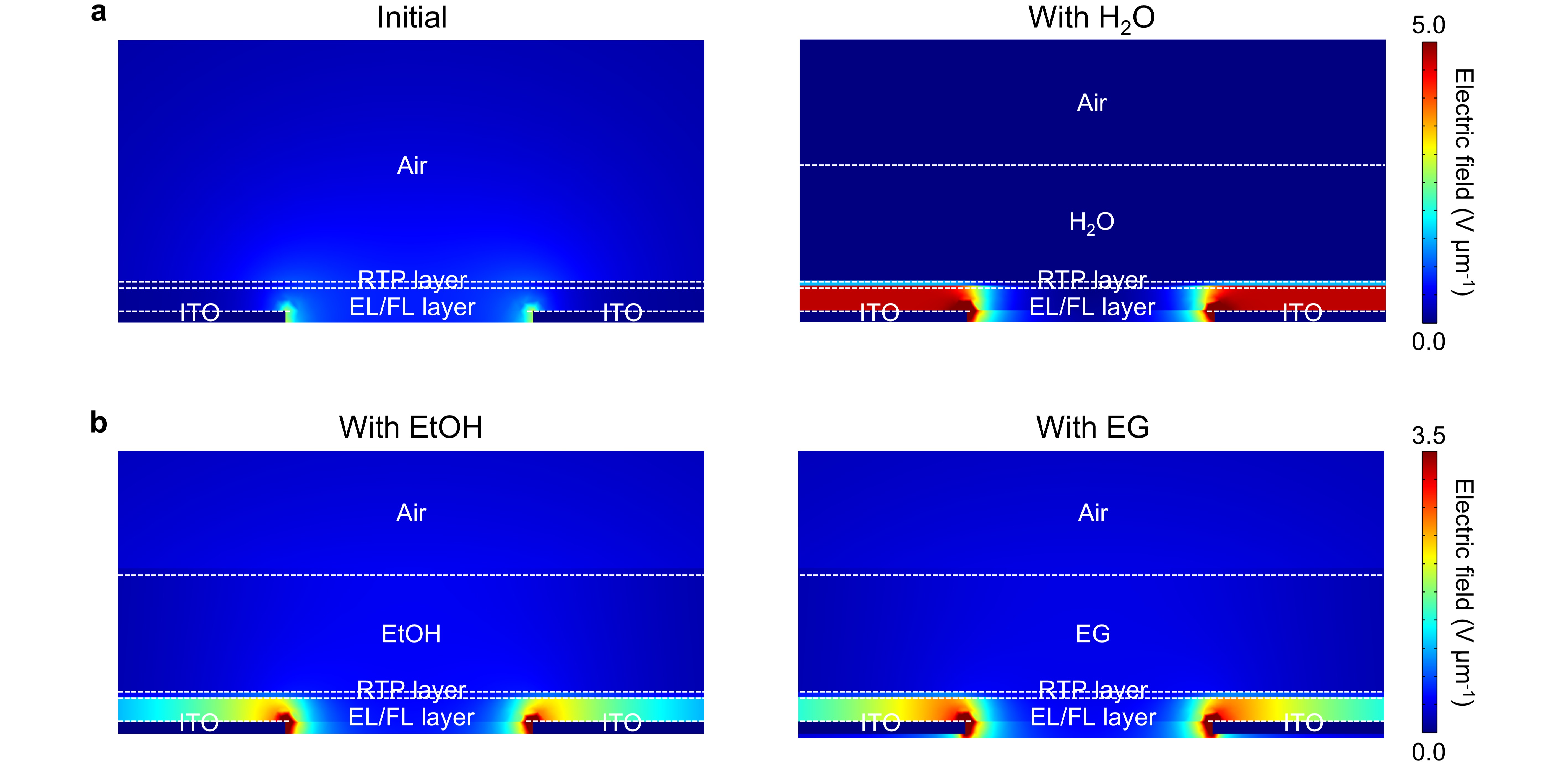


**Fig. S1** Electric field distribution of RE-TriLED. **a**, **b** Numerical simulations of electric field distribution of RE-TriLED with and without H_2_O (**a**) and with EtOH or EG (**b**) acting as a polar electrode bridge, respectively

**
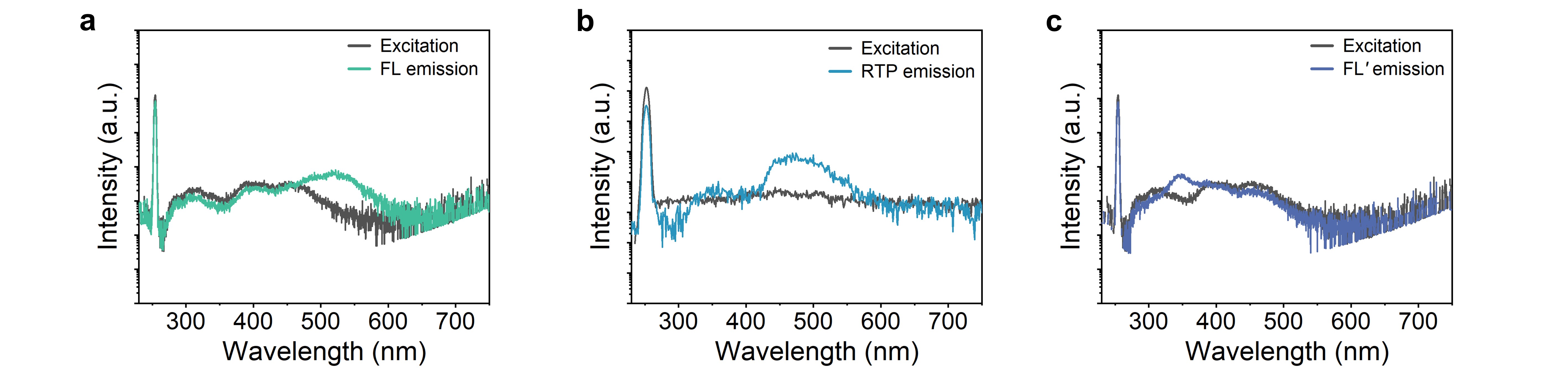
**

**Fig. S2** Excitation and emission spectra for photoluminescence quantum yield measurements. **a**-**c** FL (**a**), RTP (**b**), and FLʹ (**c**) emission spectra together with 254 nm excitation spectra


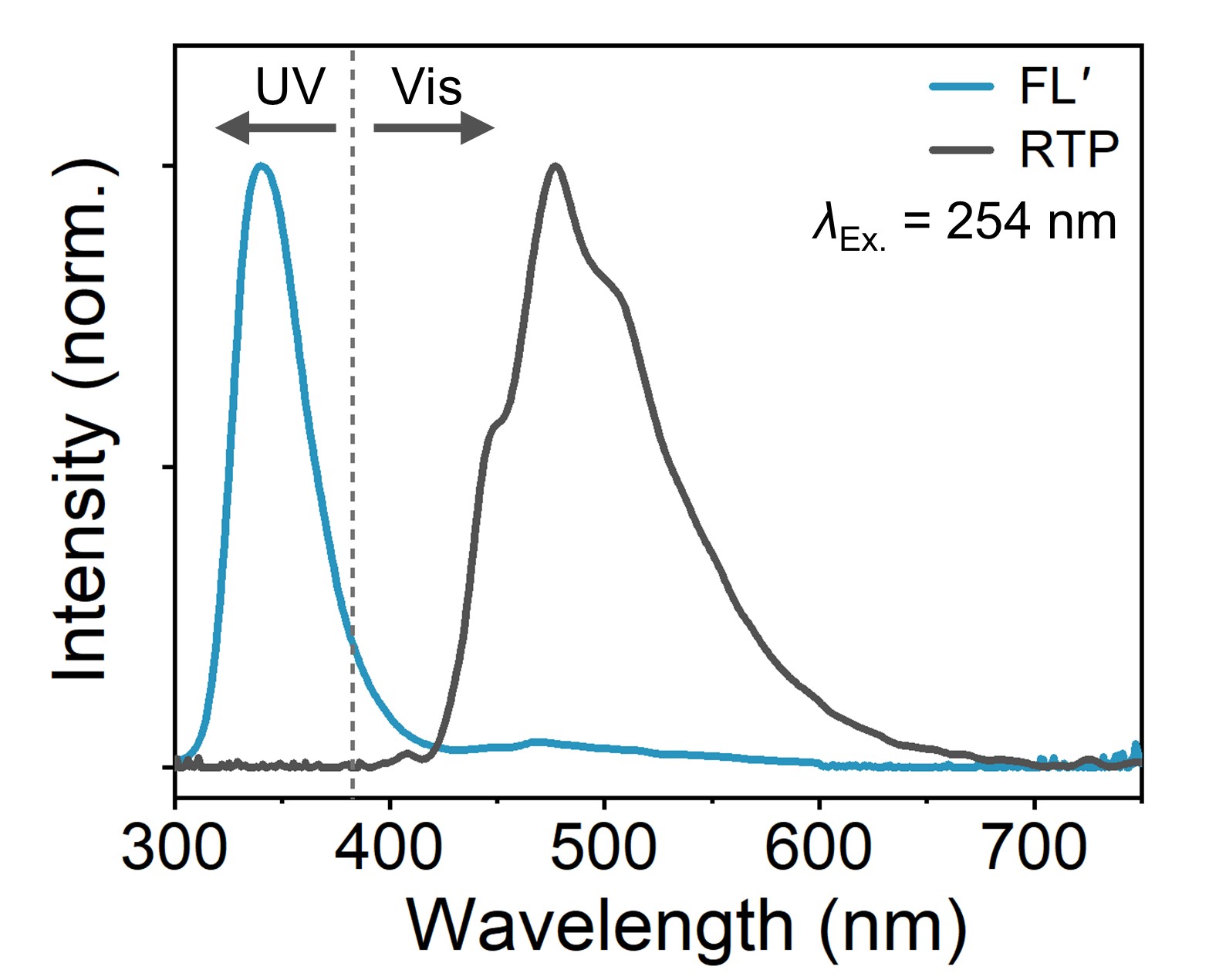


**Fig. S3** Emission spectra of RTP film with and without H_2_O exposure. Normalized spectra of FL′ (with H_2_O) and RTP emission (without H_2_O) from the RTP film under 254 nm excitation


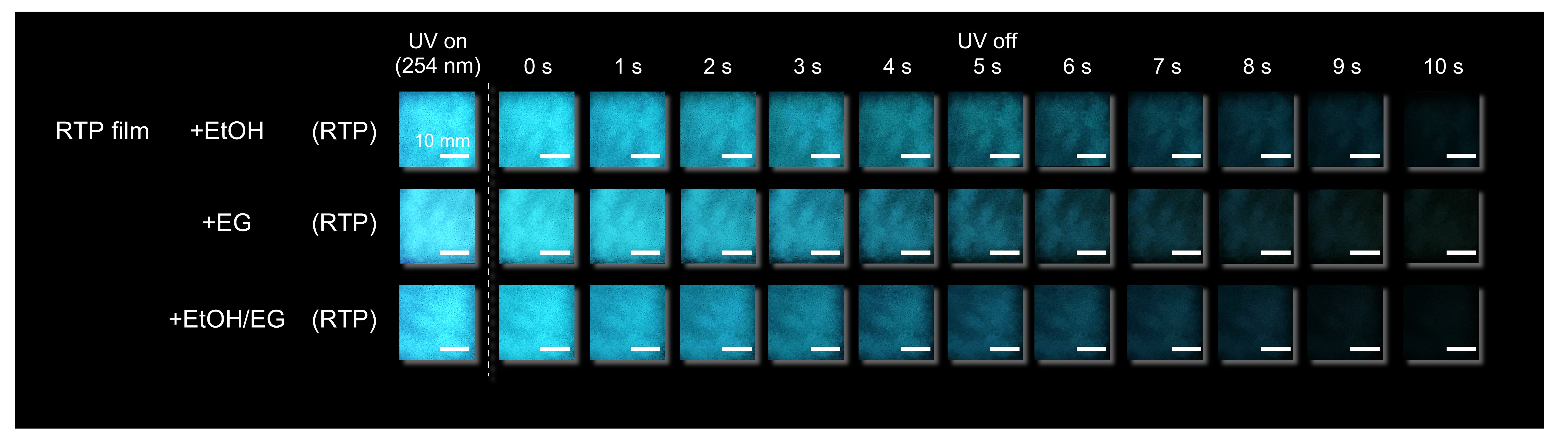


**Fig. S4** RTP performance with exposure to different polar liquids. Photographs of RTP films under 254 nm UV irradiation and after removal of UV lamp. RTP emission is retained when exposed to EtOH, EG, or EtOH/EG (polar liquids other than H_2_O)


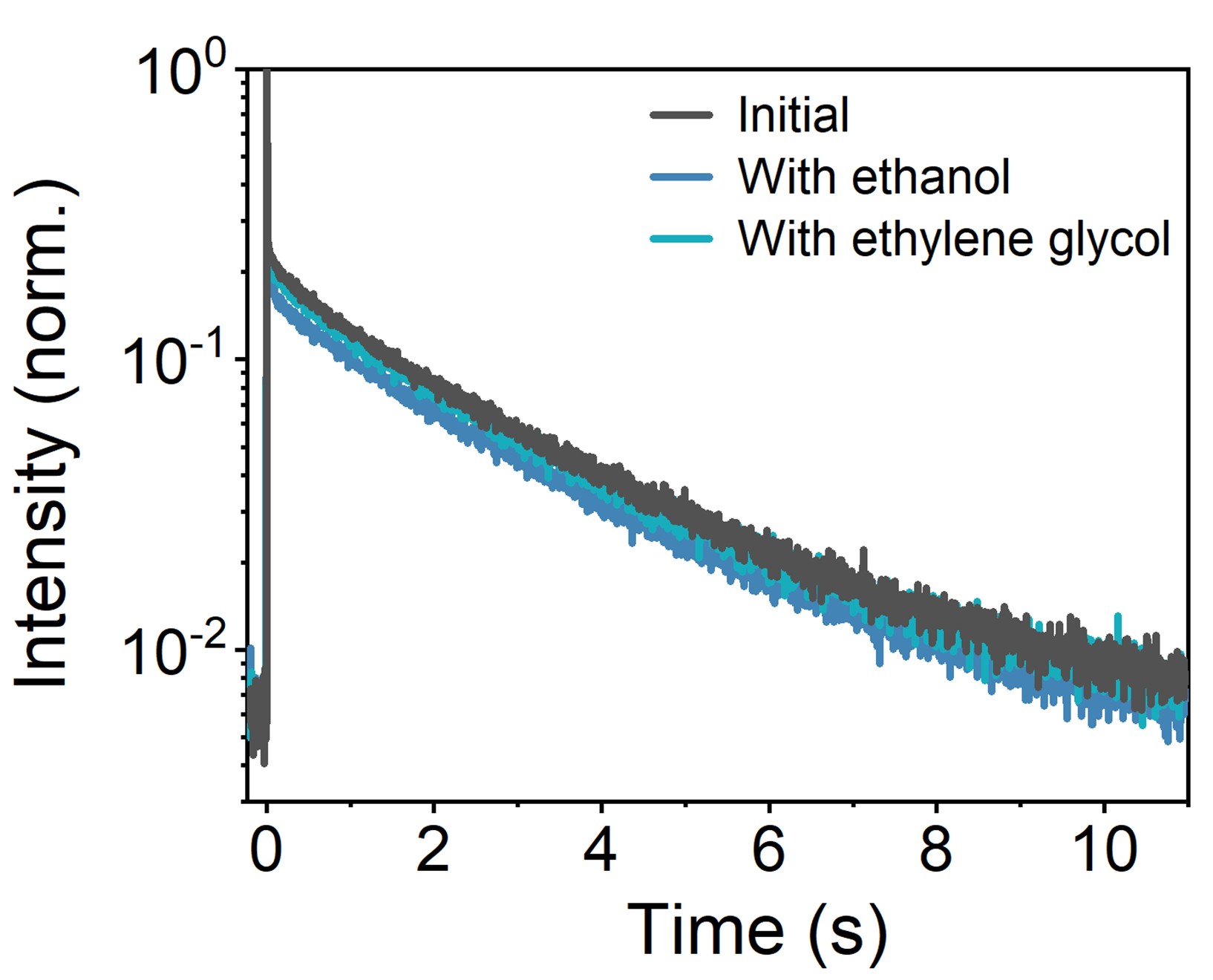


**Fig. S5** Time-resolved photoluminescence decay profiles with exposure to different polar liquids. Profiles of an RTP film at its initial state, and upon exposure to ethanol or ethylene glycol

**
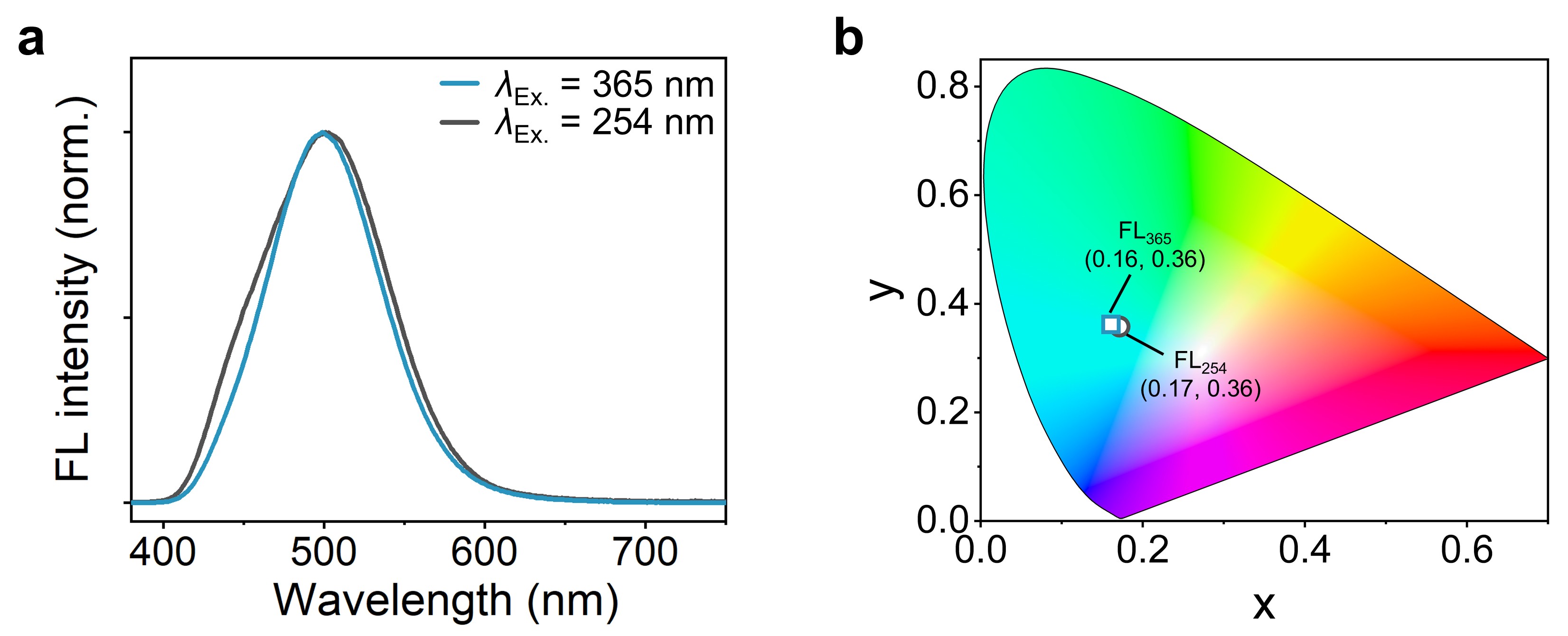
**

**Fig. S6** Comparison of FL emission under different excitation wavelengths. **a**, **b** Normalized FL emission spectra (**a**) and CIE coordinates (**b**) under 365 and 254 nm excitation wavelength


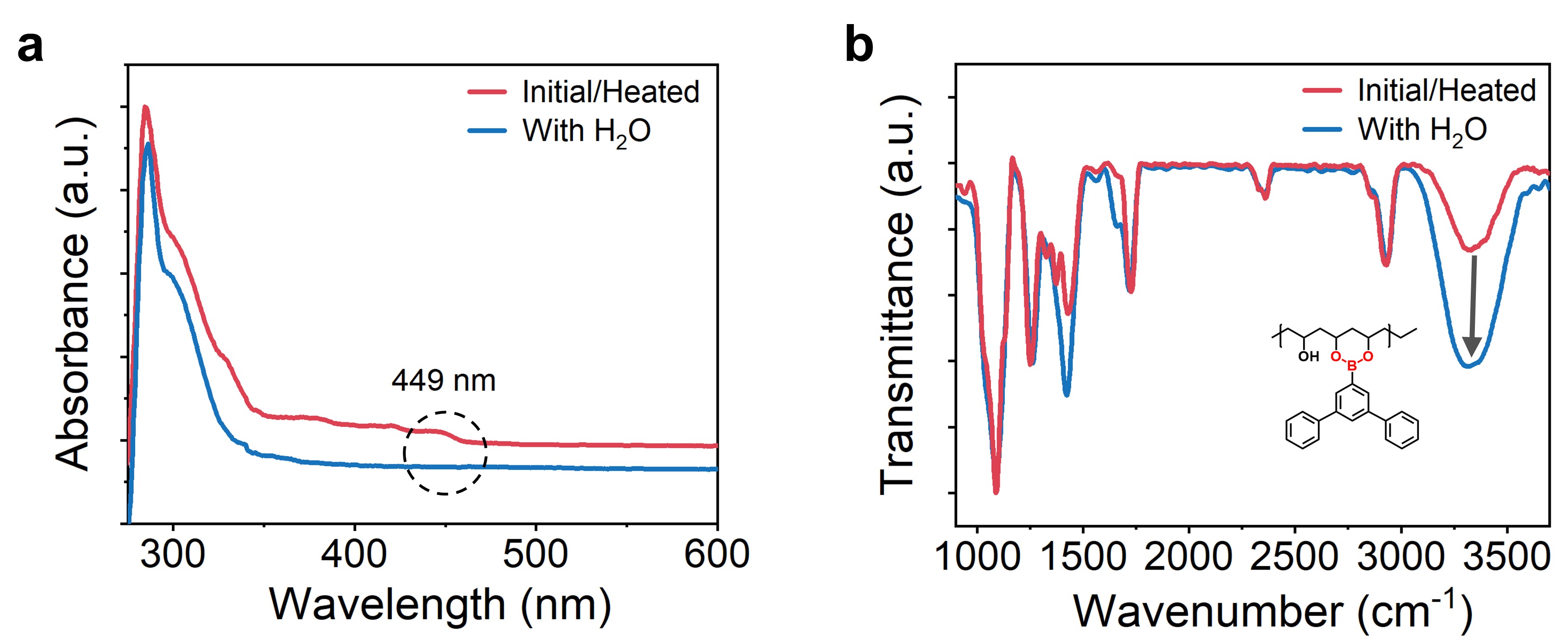


**Fig. S7** Spectral differences between RTP film with and without water exposure. **a**, **b** UV-Vis absorption spectra (**a**) and FTIR spectra (**b**) of an RTP film at initial/heated state or upon H_2_O exposure. The inset in **b** is a chemical structure of DPP-BOH-PVA with a covalent bond


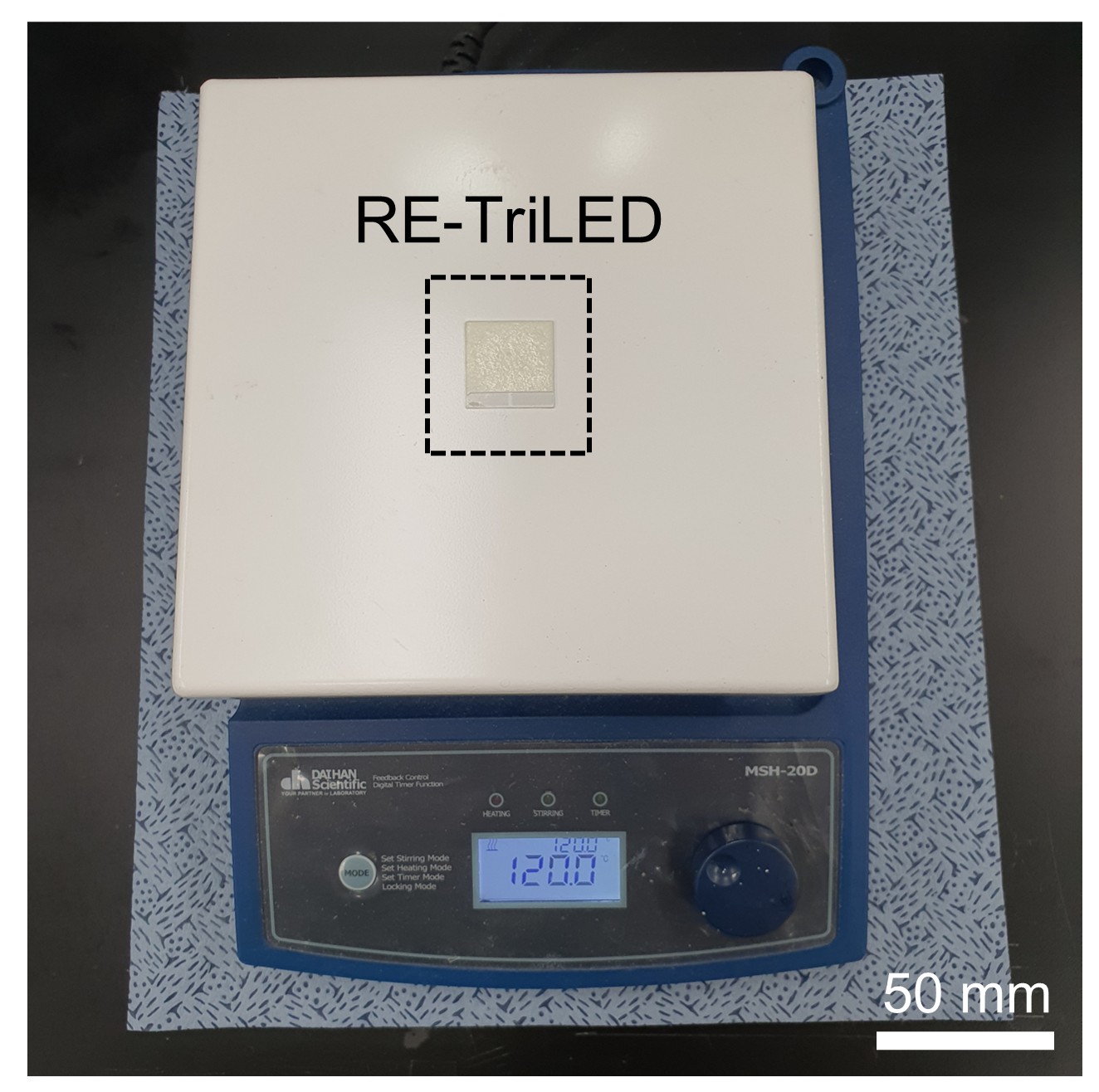


**Fig. S8** Method of heat exposure for RTP restoration of RE-TriLED. Photograph of a RE-TriLED placed on a 120 °C hotplate to remove water

**
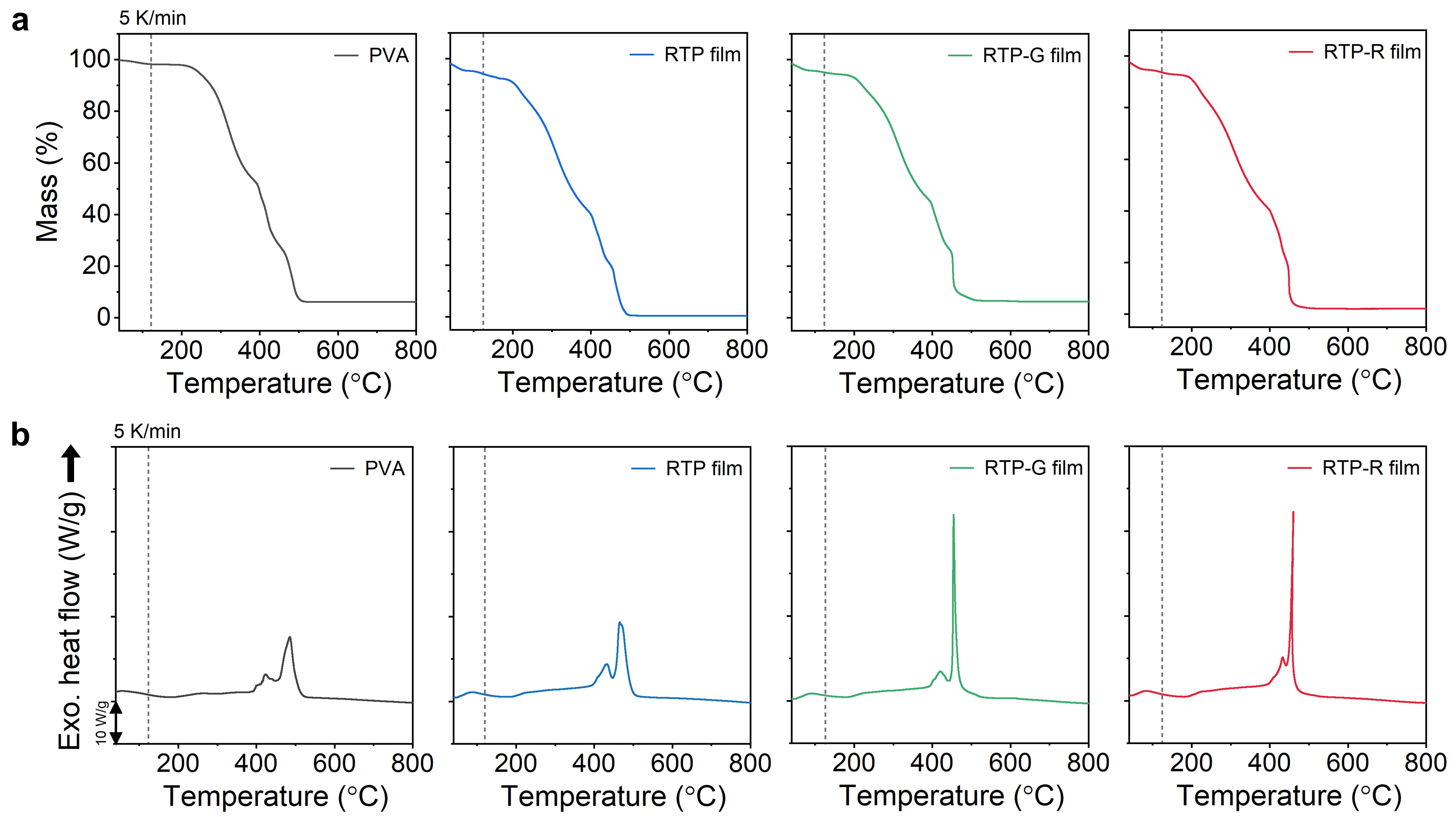
**

**Fig. S9** Evaluation of thermal stability of the RTP layer. **a**, **b** TGA (**a**) and DSC (**b**) traces obtained from PVA, RTP, RTP-G, and RTP-R films during continuous heating with a heating rate of 5 K min^-1^. The RE-TriLED was exposed to temperatures under the short-dashed lines (120 °C), which enabled reversible/rewritable operation without thermal decomposition

**
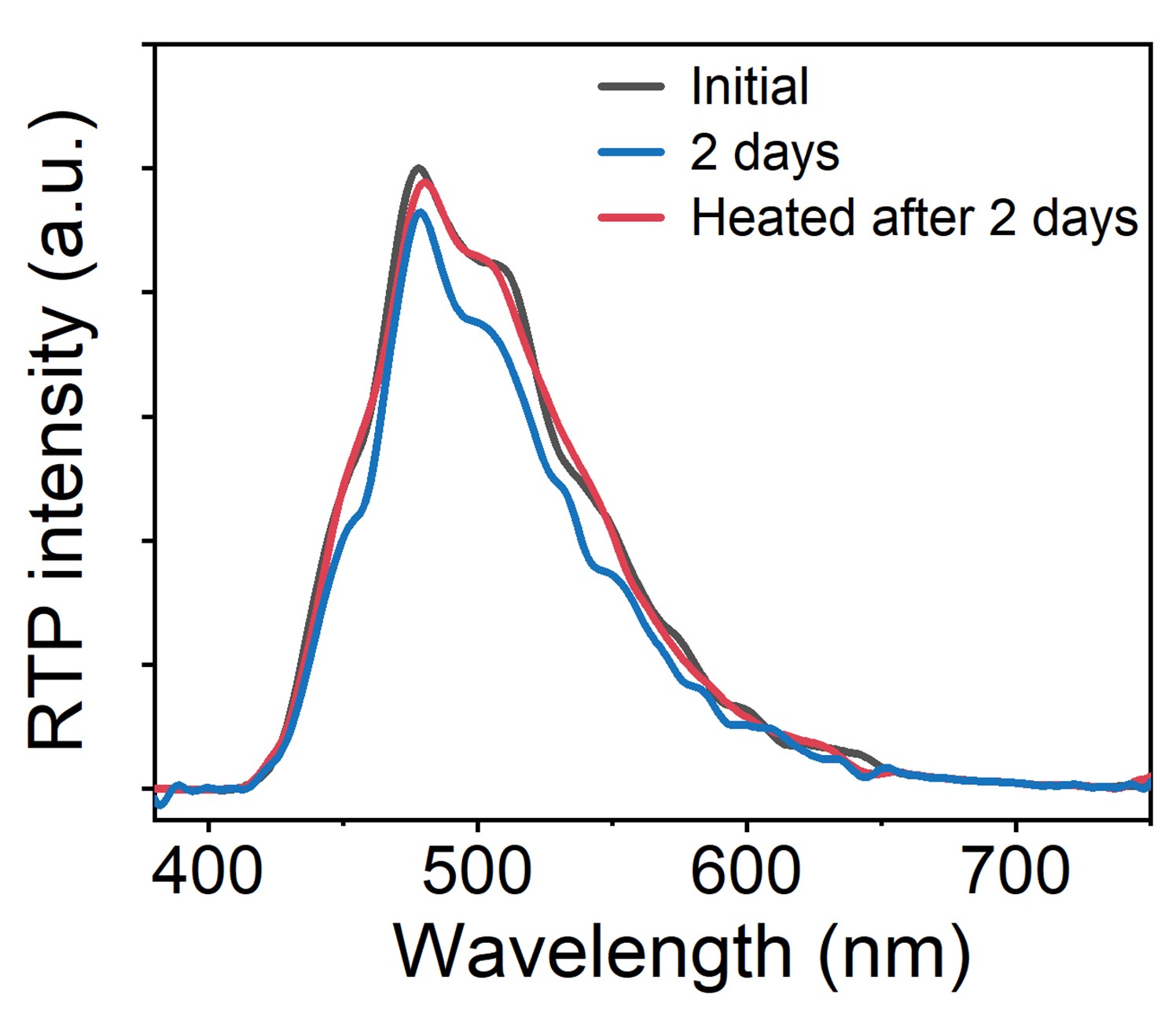
**

**Fig. S10** Performance of RTP film by time. Comparison of RTP spectra of an RTP film at its initial state, after 2 days under ambient conditions, and heating the film after 2 days


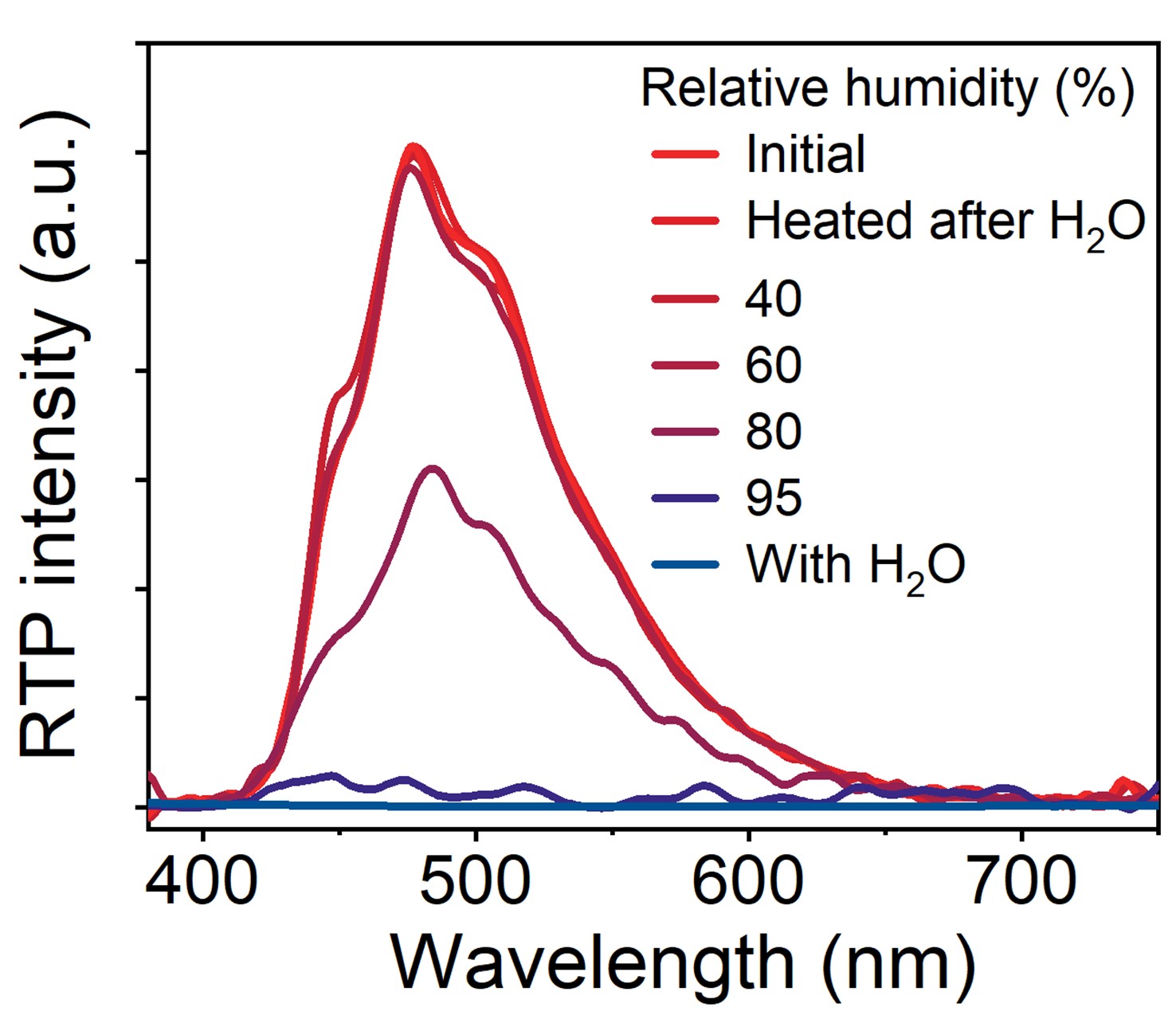


**Fig. S11** RTP characteristics under different relative humidity conditions. RTP spectra of an RTP film under various relative humidity conditions together with the spectra upon H_2_O and heat exposure


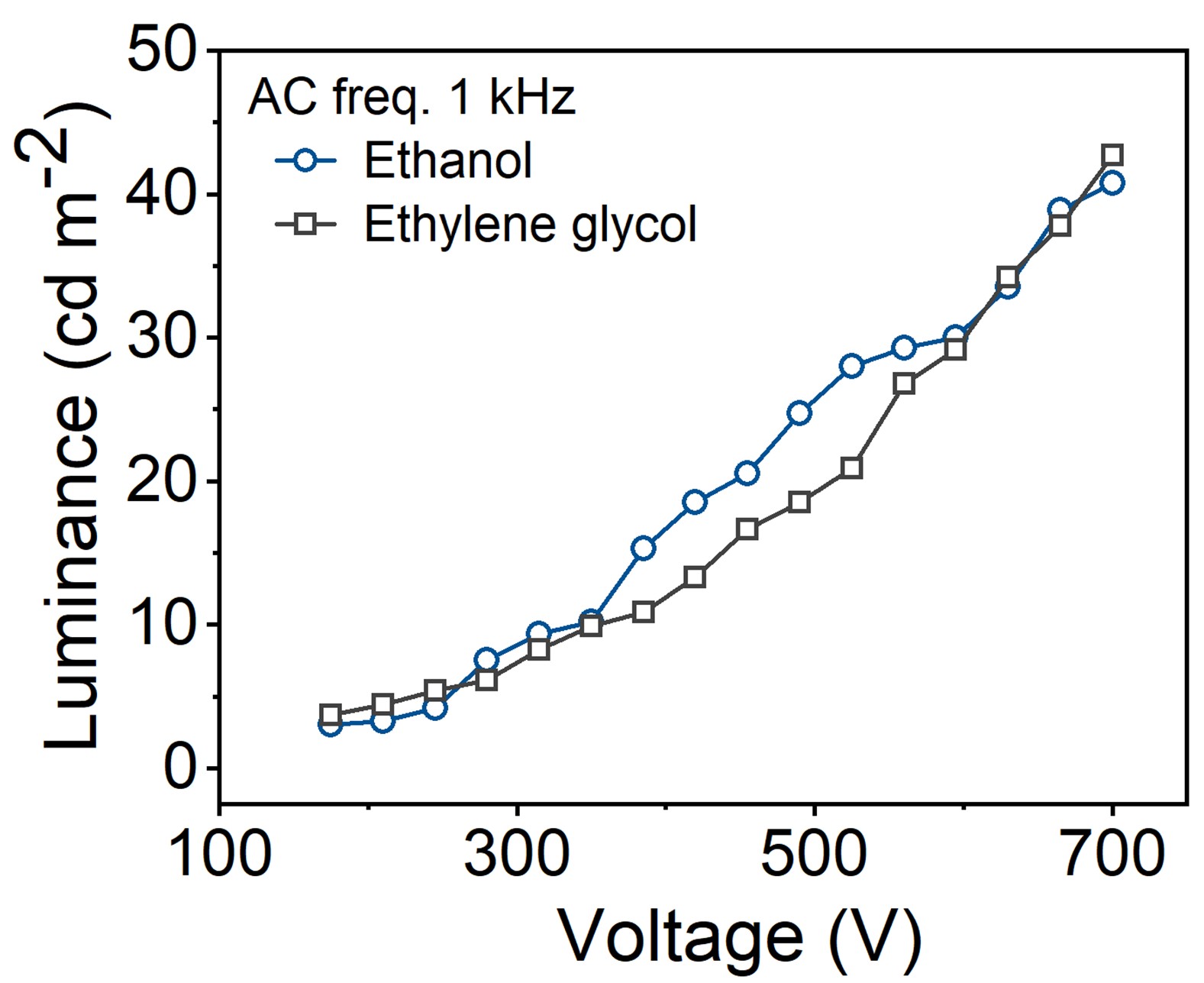


**Fig. S12** EL performance of RE-TriLED with different polar liquids. Luminance–voltage curves of a RE-TriLED polar-bridged by ethanol or ethylene glycol


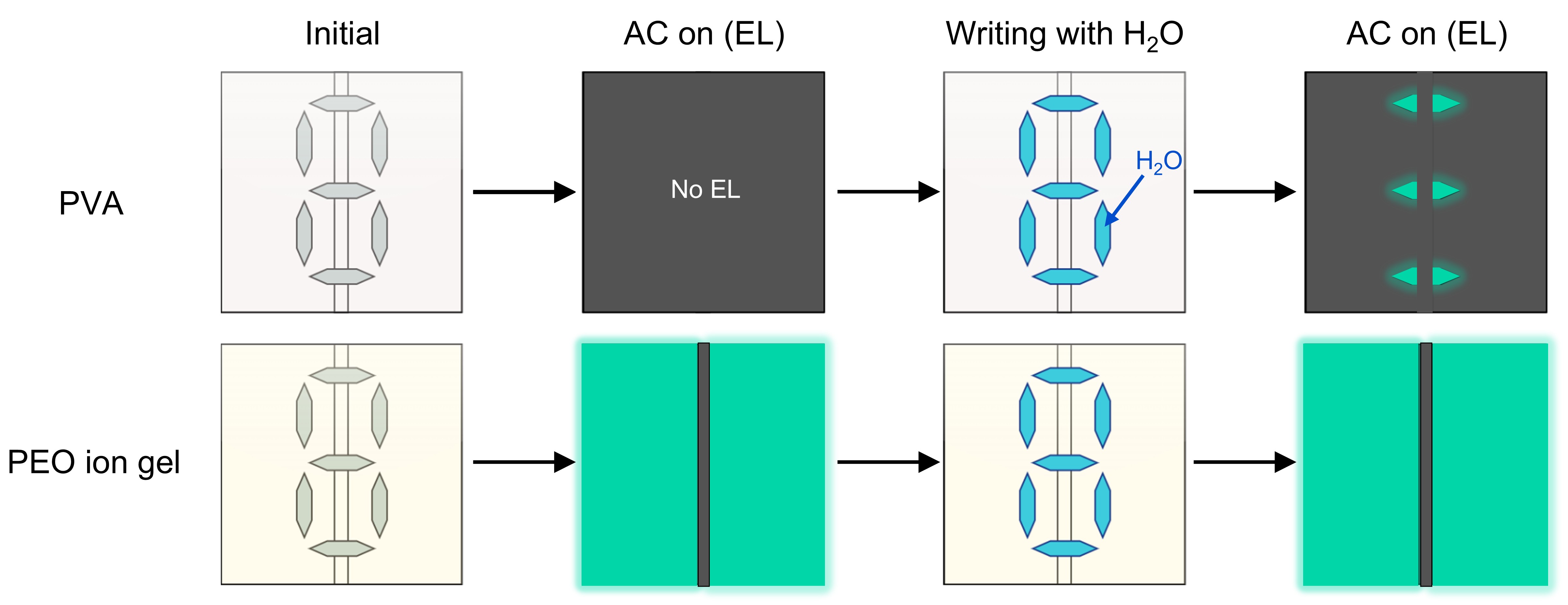


**Fig. S13** Comparison of different protective layers of the RE-TriLED. Schematic illustration comparing EL emission of a RE-TriLED when PVA and PEO ion gel is used as a protective layer. RE-TriLED with a PVA layer emits EL only when H_2_O is added, and the emission area is only where H_2_O crosses the gap between the two electrodes. RE-TriLED with a PEO ion gel layer emits EL regardless of the presence of H_2_O


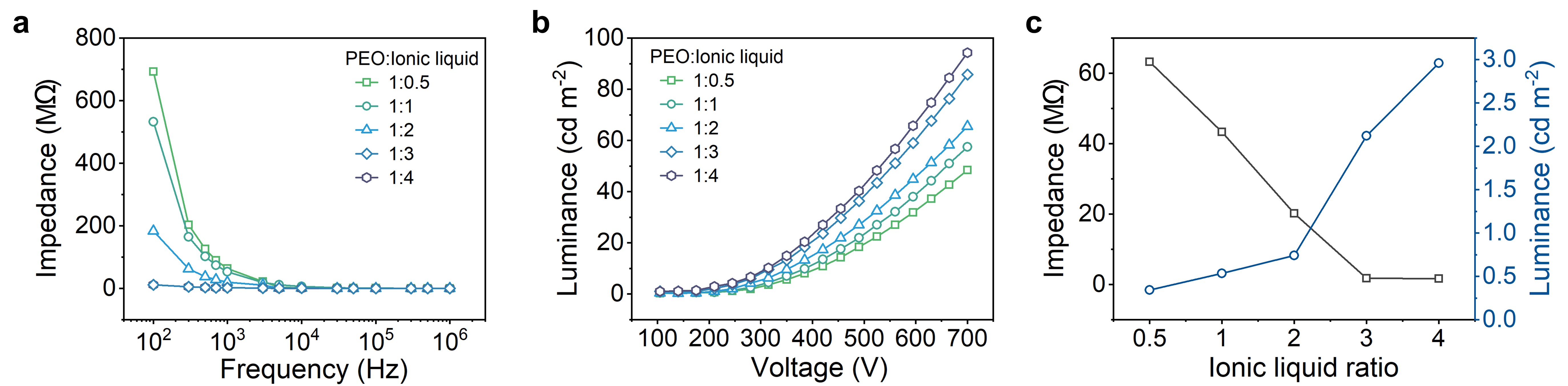


**Fig. S14** EL characteristics with different composition ratios of PEO ion gel. **a**, **b** Impedance (**a**) and luminance–voltage (**b**) curves of a RE-TriLED as a function of PEO to ionic liquid ratio of the PEO ion gel acting as a polar electrode bridge. **c** Impedance and luminance characteristics at AC 1 kHz and 175 V for different amounts of ionic liquid of the PEO ion gel (ionic liquid/PEO)


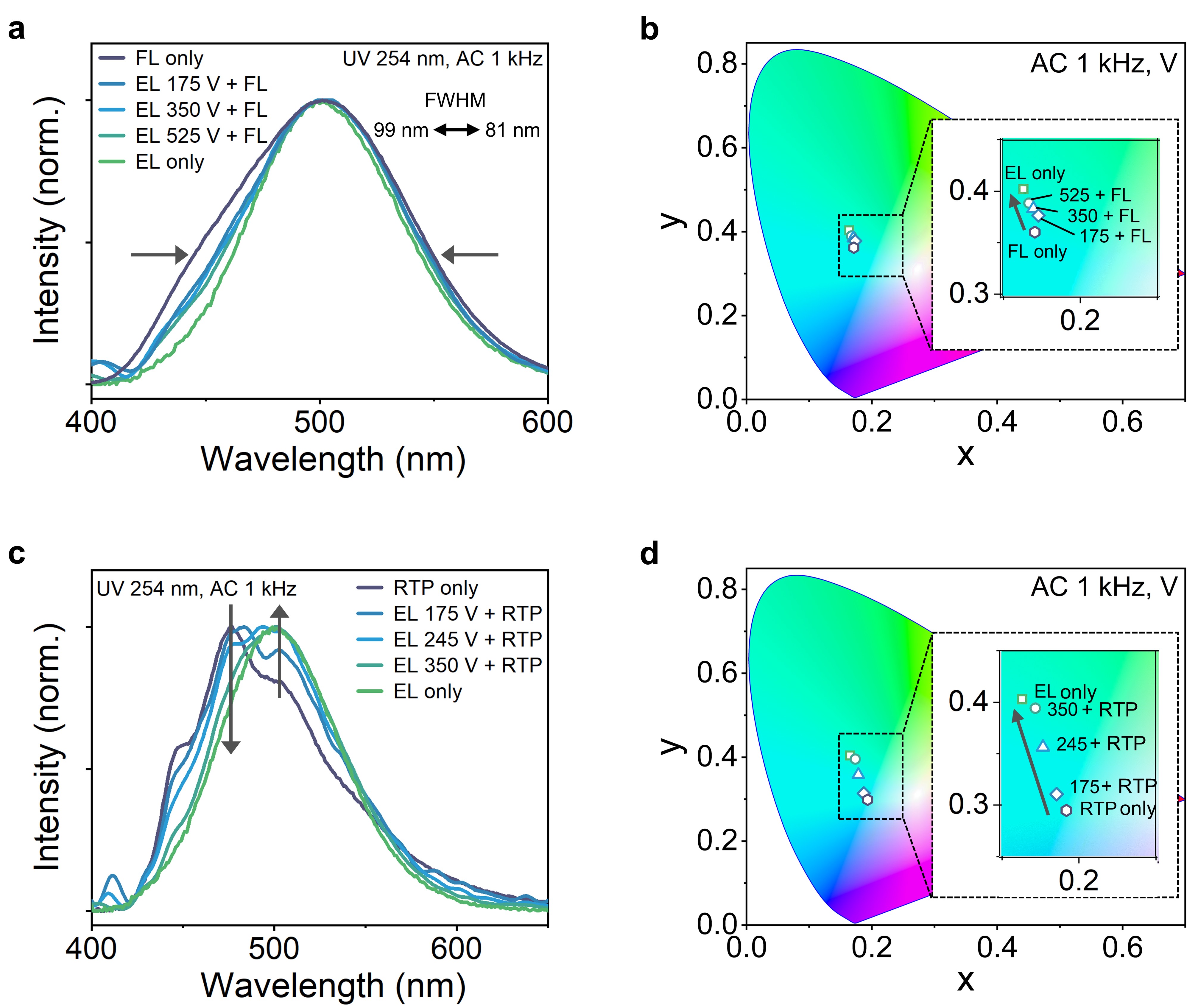


**Fig. S15** Spectra and CIE coordinates of RE-TriLED emitting two modes simultaneously. **a**, **b** Normalized spectra (**a**) and CIE coordinates (**b**) of FL and EL emission as a function of AC voltage. **c**, **d** Normalized spectra (**c**) and CIE coordinates (**d**) of RTP and EL emission as a function of AC voltage. The insets in **b** and **d** show a magnified view of the CIE coordinates. As the voltage increases, the spectrum and CIE coordinate shifts toward “EL only”


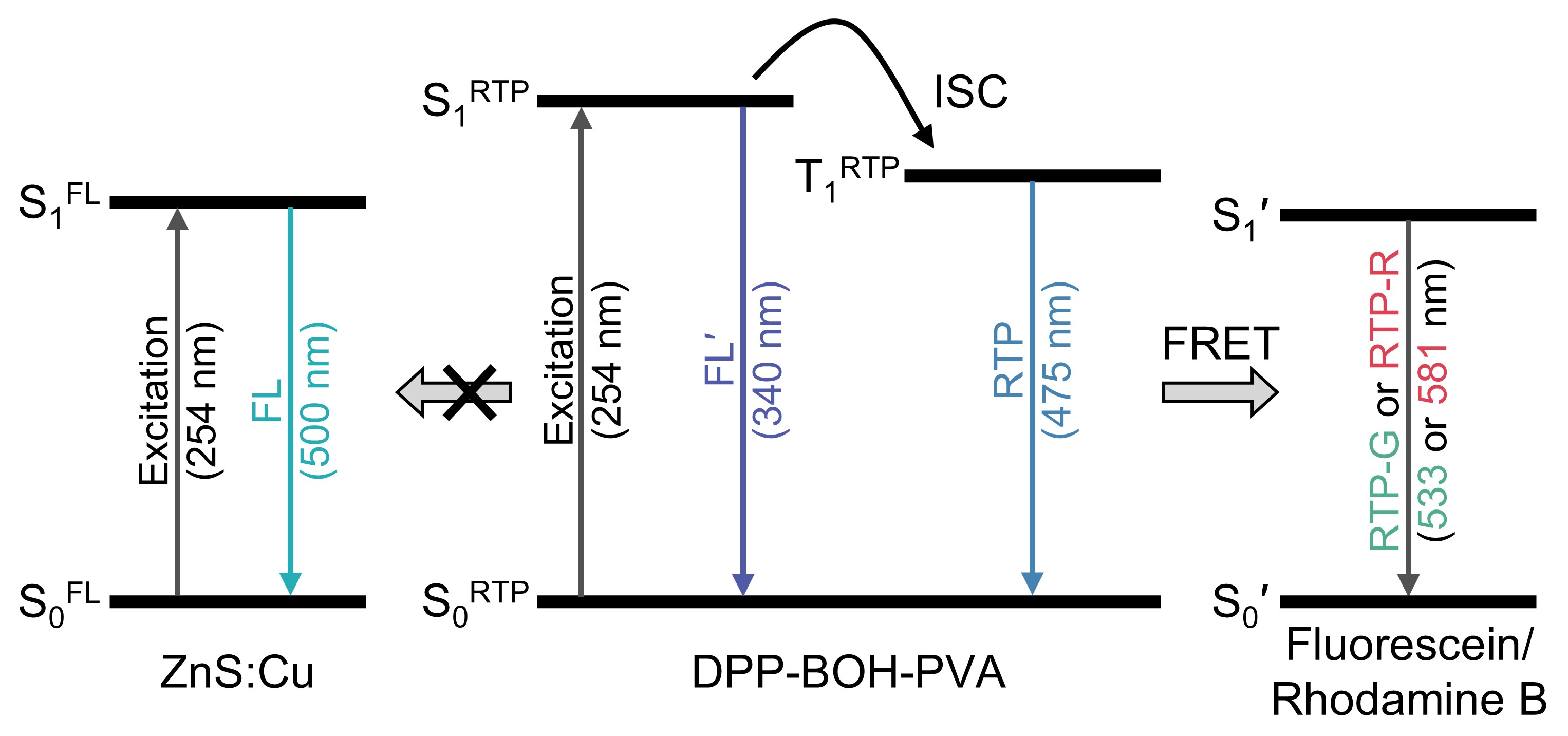


**Fig. S16** Diagram of afterglow mechanism for RTP-G and RTP-R films. Simplified Jablonski diagram showing Förster-resonance energy transfer (FRET) from an energy donor, DPP-BOH-PVA, to an energy acceptor (fluorescein or rhodamine B). Here, energy transfer does not occur from the RTP layer to the EL/FL layer (ZnS:Cu). ISC indicates intersystem crossing


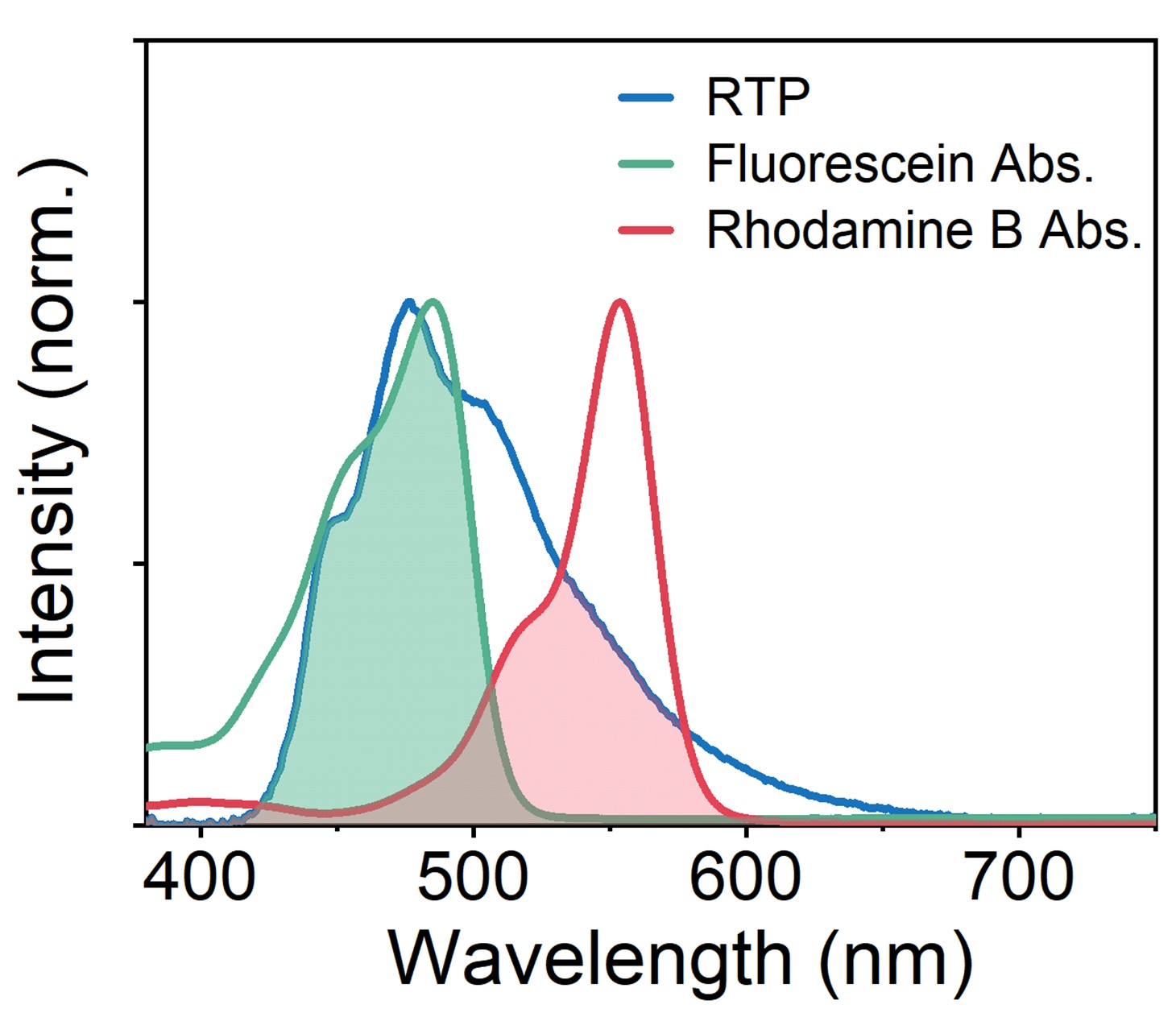


**Fig. S17** Spectral overlap between RTP emission and absorbance of fluorescent dyes. RTP emission spectrum and UV-Vis absorption spectra of fluorescein and rhodamine B. The green and red areas indicate spectral overlap between the RTP spectrum and absorption spectra of fluorescein and rhodamine B, respectively


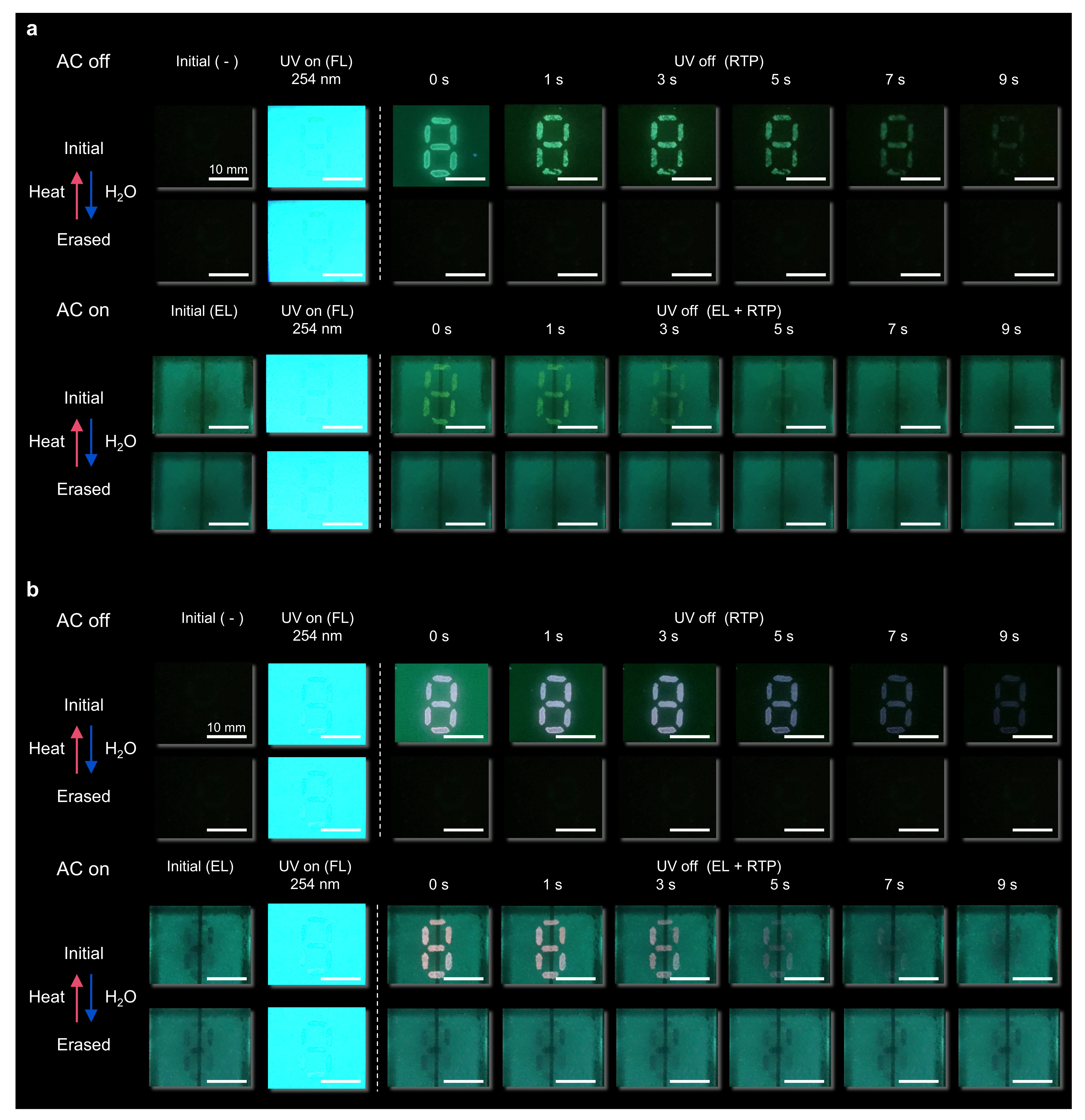


**Fig. S18** Mode-selective RE-TriLED with reversible RTP-G and RTP-R. **a**, **b** Photographs of RE-TriLEDs with a patterned RTP-G layer (**a**) or RTP-R layer (**b**) under UV irradiation and after removal of UV lamp with and without AC field. The RE-TriLEDs were also exposed to either H_2_O or heat for reversible RTP emission. The two RE-TriLEDs were fabricated through the procedure described in Fig. 3a


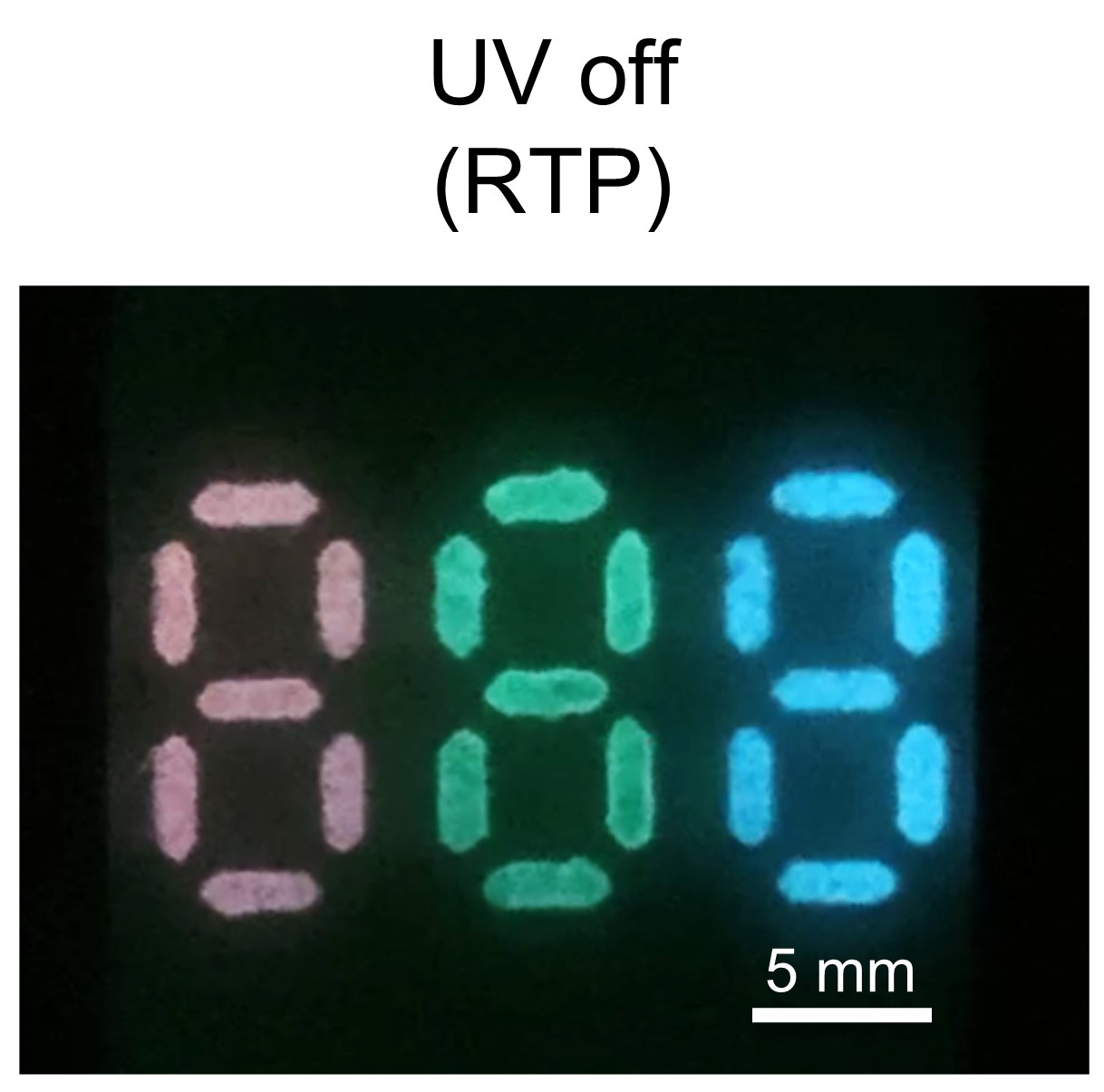


**Fig. S19** RTP emission of full visible RE-TriLED. Photograph of a full visible RE-TriLED only after removal of UV lamp


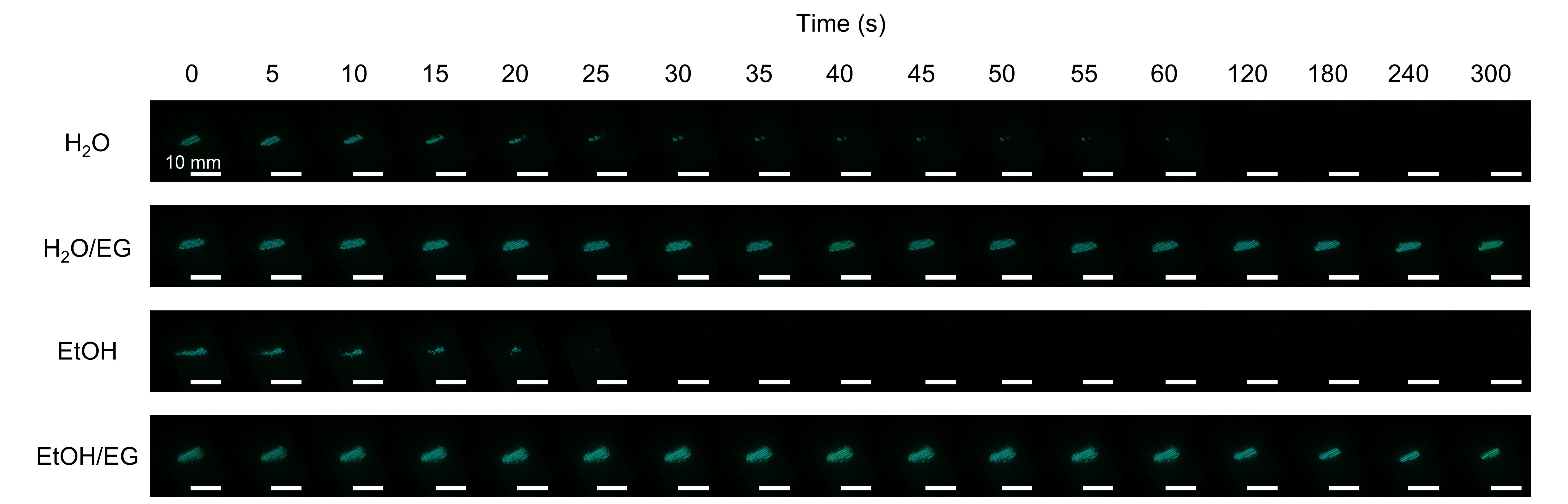


**Fig. S20** Photographs of EL emission over time. Photographs of EL emission over time with H_2_O, H_2_O/EG, EtOH, and EtOH/EG acting as a polar electrode bridge


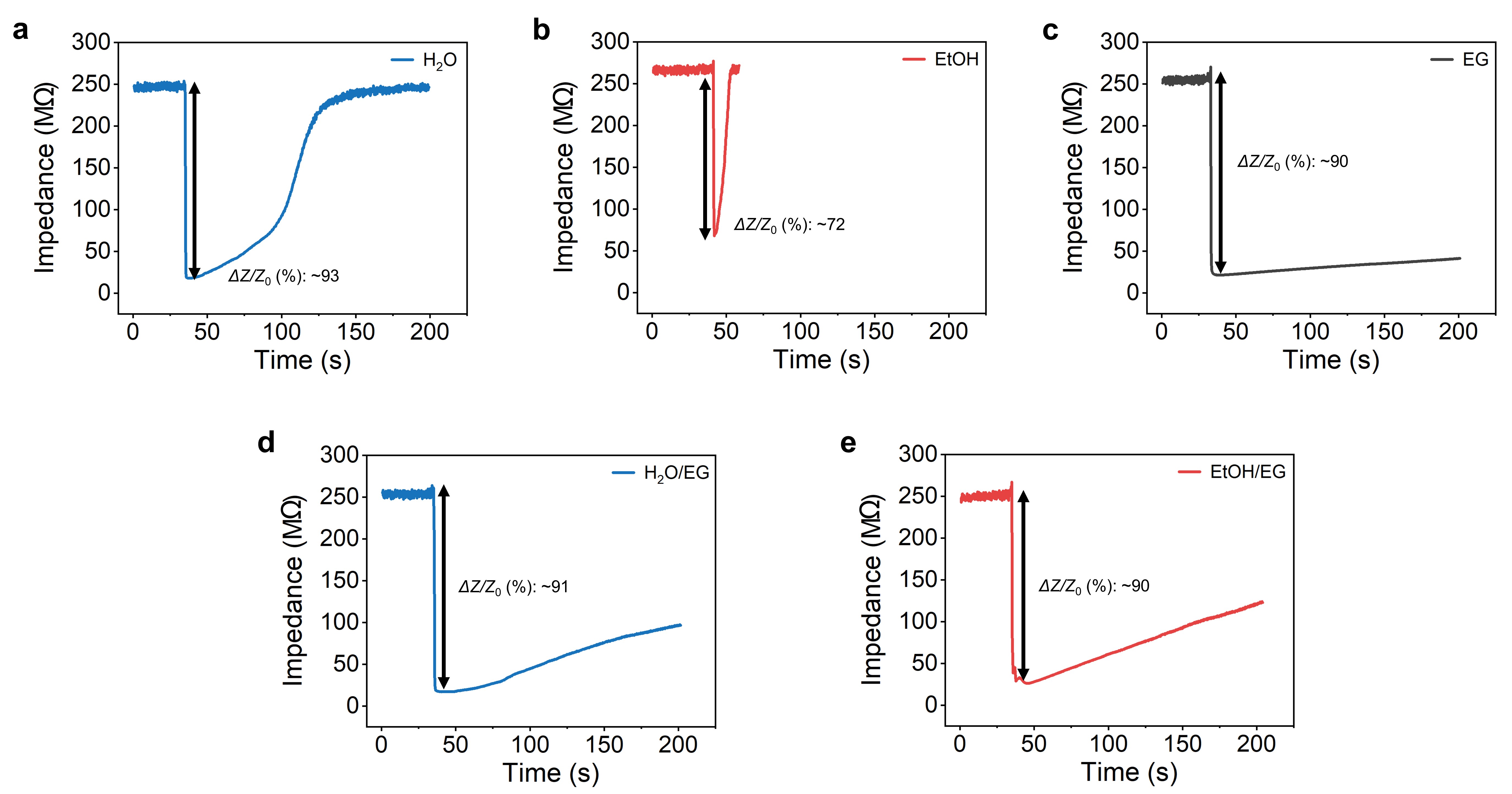


**Fig. S21** EL retention properties of different polar liquids. **a**-**e** Impedance curves when H_2_O (**a**), EtOH (**b**), EG (**c**), H_2_O/EG (**d**), and EtOH/EG (**e**) are dropped on a RE-TriLED, with each of the polar liquids acting as a polar electrode bridge
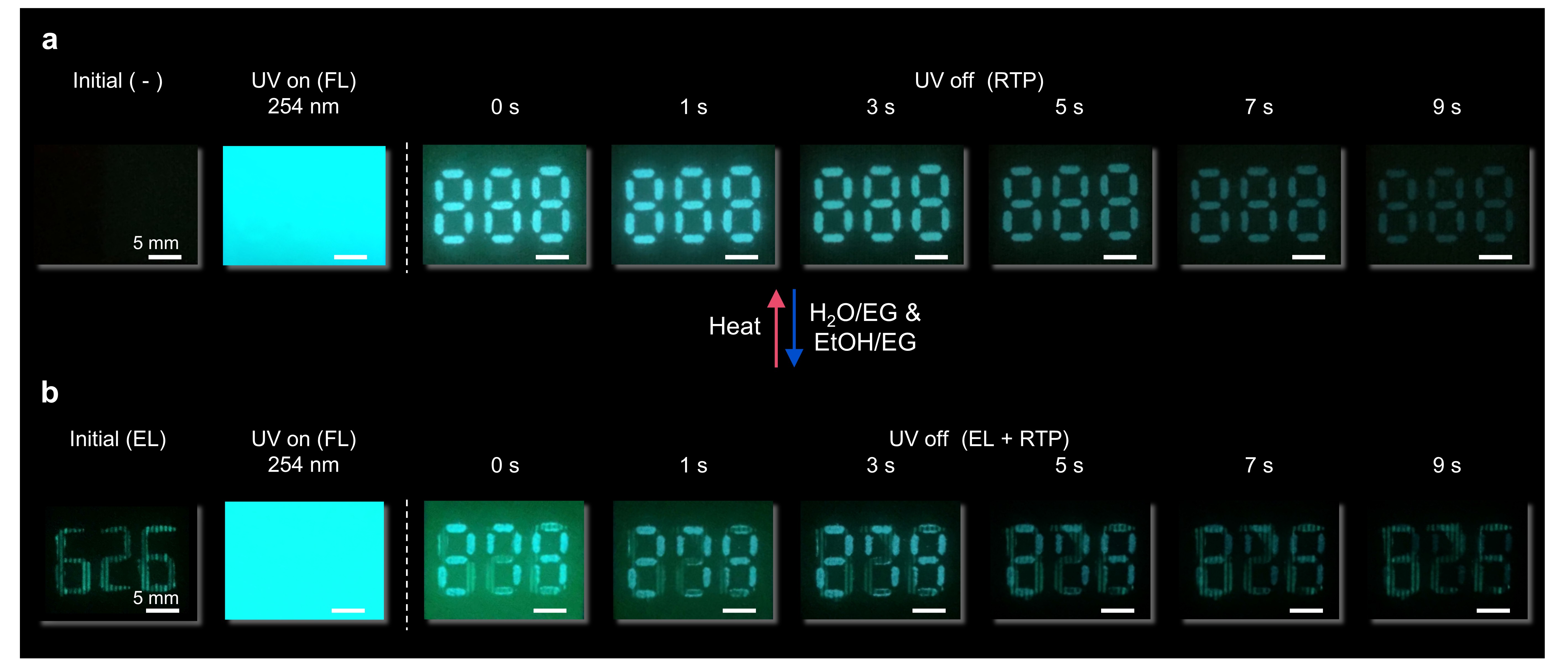


**Fig. S22** Photographs of multilevel encryption with a patterned RE-TriLED. **a**, **b** Photographs of a patterned RE-TriLED under UV irradiation and after removal of UV lamp before (**a**) and after exposure (**b**) to H_2_O/EG and EtOH/EG mixtures. AC field is also applied for **b**


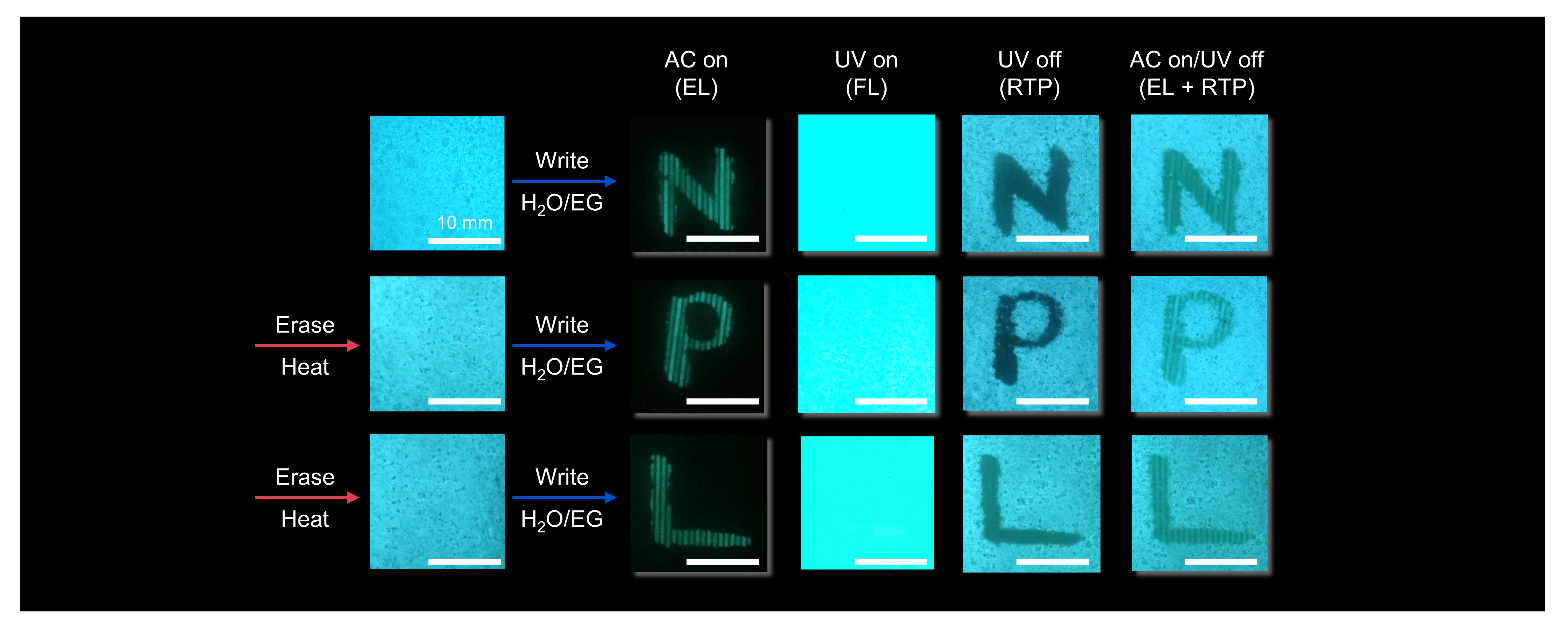


**Fig. S23** Repetitive writing and erasing on RE-TriLED. Photographs of a nonpatterned RE-TriLED showing different letters by its rewritability

**Movie S1** Response of RTP film to different polar liquids and UV exposure

**Movie S2** Mode-selective RE-TriLED with reversible RTP

**Movie S3** RE-TriLED with full visible RTP

**Movie S4** Rewritable RE-TriLED with independent EL and RTP encryption

**Supplementary References**

1. W. Jiang, S. Lee, G. Zan, K. Zhao, C. Park, Alternating current electroluminescence for human-interactive sensing displays. Adv. Mater. **36**, 2304053 (2023). <https://doi.org/10.1002/adma.202304053>
2. G. F. Alfrey, J. B. Taylor, Electroluminescence in single crystals of zinc sulphide. Proc. Phys. Soc. B **68**, 775 (1955). <https://doi.org/10.1088/0370-1301/68/10/310>
3. S. Ishaq, F. Kanwal, S. Atiq, M. Moussa, U. Azhar et. al., Dielectric and impedance spectroscopic studies of three phase graphene/titania/poly(vinyl alcohol) nanocomposite films. Results Phys. **11**, 540–548 (2018). <https://doi.org/10.1016/j.rinp.2018.09.049>
4. M. Prego, O. Cabeza, E. Carballo, C. F. Franjo, E. Jiménez, Measurement and interpretation of the electrical conductivity of 1-alcohols from 273 K to 333 K. J. Mol. Liq. **89**, 233–238 (2000). <https://doi.org/10.1016/S0167-7322(00)90016-3>
5. U. Mayer, V. Gutmann, W. Gerger, The acceptor number — A quantitative empirical parameter for the electrophilic properties of solvents. Monatsh. Chem. **106**, 1235–1257 (1975). <https://doi.org/10.1007/BF00913599>
6. L. Besra, M. Liu, A review on fundamentals and applications of electrophoretic deposition (EPD). Prog. Mater. Sci. **52**, 1–61 (2007). <https://doi.org/10.1016/j.pmatsci.2006.07.001>
7. G. D. Pasquale, S. Graziani, F. G. Messina, A. Pollicino, R. Puglisi et. al., An investigation of the structure–property relationships in ionic polymer polymer composites (IP^2^Cs) manufactured by polymerization *in situ* of PEDOT/PSS on Nafion^®^117. Smart Mater. Struct. **23**, 035018 (2014). <https://doi.org/10.1088/0964-1726/23/3/035018>
8. B. Yang, Y. Zhao, M. U. Ali, J. Ji, H. Yan et. al., Asymmetrically enhanced coplanar-electrode electroluminescence for information encryption and ultrahighly stretchable displays. Adv. Mater. **34**, 2201342 (2022). <https://doi.org/10.1002/adma.202201342>
9. S. Zhang, Y. Zhu, Y. Xia, K. Liu, S. Li et. al., Wearable integrated self-powered electroluminescence display device based on all-in-one MXene electrode for information encryption. Adv. Funct. Mater. **33**, 2307609 (2023). <https://doi.org/10.1002/adfm.202307609>
10. G. Lee, M. Kong, D. Park, J. Park, U. Jeong, Electro-photoluminescence color change for deformable visual encryption. Adv. Mater. **32**, 1907477 (2020). <https://doi.org/10.1002/adma.201907477>
11. J. Oh, D. Baek, T. K. Lee, D. Kang, H. Hwang et. al., Dynamic multimodal holograms of conjugated organogels via dithering mask lithography. Nat. Mater. **20**, 385–394 (2021). <https://doi.org/10.1038/s41563-020-00866-4>
12. H. Han, J. W. Oh, H. Lee, S. Lee, S. Mun et. al., Rewritable photoluminescence and structural color display for dual‐responsive optical encryption. Adv. Mater. **36**, 2310130 (2024). <https://doi.org/10.1002/adma.202310130>
13. D. Li, Y. Yang, J. Yang, M. Fang, B. Z. Tang et. al., Completely aqueous processable stimulus responsive organic room temperature phosphorescence materials with tunable afterglow color. Nat. Commun. **13**, 347 (2022). <https://doi.org/10.1038/s41467-022-28011-6>
14. D. Li, J. Yang, M. Fang, B. Z. Tang, Z. Li, Stimulus-responsive room temperature phosphorescence materials with full-color tunability from pure organic amorphous polymers. Sci. Adv. **8**, eabl8392 (2022). <https://doi.org/10.1126/sciadv.abl8392>
15. Z. Wang, Y. Zhang, C. Wang, X. Zheng, Y. Zheng et. al., Color-tunable polymeric long-persistent luminescence based on polyphosphazenes. Adv. Mater. **32**, 1907355 (2020). <https://doi.org/10.1002/adma.201907355>
16. X. Wang, G. Pan, H. Ren, J. Li, B. Xu et. al., Reversible photoswitching between fluorescence and room temperature phosphorescence by manipulating excited state dynamics in molecular aggregates. Angew. Chem. Int. Ed. **61**, e202114264 (2022). <https://doi.org/10.1002/anie.202114264>
17. G. Zhou, Y. Mao, J. Zhang, Q. Ren, M. S. Molokeev et. al., Dynamic phosphorescence/fluorescence switching in hybrid metal halides toward time-resolved multi-level anti-counterfeiting. Adv. Mater. **32**, 1907355 (2020). <https://doi.org/10.1002/adfm.202413524>
18. J. W. Oh, S. Lee, H. Han, O. Allam, J. I. Choi et. al., Dual-light emitting 3D encryption with printable fluorescent-phosphorescent metal-organic frameworks. Light Sci. Appl. **12**, 226 (2023). <https://doi.org/10.1038/s41377-023-01274-4>
19. Q. Wang, B. Lin, M. Chen, C. Zhao, H. Tian et. al., A dynamic assembly-induced emissive system for advanced information encryption with time-dependent security. Nat. Commun. **13**, 4185 (2022). <https://doi.org/10.1038/s41467-022-31978-x>
20. P. She, Y. Ma, Y. Qin, M. Xie, F. Li et. al., Dynamic luminescence manipulation for rewritable and multi-level security printing. Matter **1**, 1644–1655 (2019). <https://doi.org/10.1016/j.matt.2019.08.016>
21. S. Xu, W. Wang, H. Li, J. Zhang, R. Chen et. al., Design of highly efﬁcient deep-blue organic afterglow through guest sensitization and matrices rigidiﬁcation. Nat. Commun. **11**, 4802 (2020). <https://doi.org/10.1038/s41467-020-18572-9>
